# Soluble bacterial lipopeptides suppress gasdermin D-associated IL-33 release in keratinocytes and atopic dermatitis in mice

**DOI:** 10.1038/s41467-026-72376-x

**Published:** 2026-05-13

**Authors:** Helen Williams, Ryo Muko, Emily Wright, Reynard Spiess, Hiroshi Matsuda, Akane Tanaka, Peter D. Arkwright, Joanne L. Pennock

**Affiliations:** 1 Lydia Becker Institute of Immunology and Inflammation, University of Manchester, Manchester, UK; 2 Laboratories of Comparative Animal Medicine, Tokyo University of Agriculture & Technology, Tokyo, Japan; 3 Manchester Institute of Biotechnology, University of Manchester, Manchester, UK

**Keywords:** Cellular microbiology, Antimicrobial responses, Atopic dermatitis

## Abstract

The rising prevalence of allergic diseases over the last century has been linked to smaller families and the shift of populations from countryside to cities, leading to reduced exposure to environmental bacteria. We previously demonstrated that *Staphylococcus aureus*-derived Second immunoglobulin-binding protein (Sbi) drives type 2 immune responses and atopic dermatitis (AD). Here we show that contrary to current dogma, soluble lipopeptides, particularly diacylated lipopeptides released by Gram-positive bacteria in their stationary phase suppress type 2 immune responses in vitro and eczema in the NC/Tnd mouse model. The immunomodulatory activity of these lipopeptides is destroyed by lipoprotein lipase. Their mechanism of immunomodulation is independent of CD14 and toll-like receptor (TLR) signaling but rather associated with inhibition of caspase/gasdermin D (GSDMD)-mediated release of the interleukin (IL)-33 alarmin from the nucleus. Our findings help to explain why exposure to environmental bacteria and topical application of bacterial commensals suppresses AD. We suggest that soluble bacterial lipopeptides could be developed into a novel class of therapeutics for treatment of allergic diseases.

## Abstract (web summary)

Here, the authors show that bacteria release immunosuppressive lipopeptides that suppress eczema and may serve as a potential therapy. This activity is independent of toll-like receptor signalling but involves gasdermin D-induced release of IL-33.

## Introduction

The interplay between pathogenic and commensal bacteria at epithelial surfaces is pivotal in maintaining health^1^. Exposure to high concentrations of microbial components such as endotoxin in unpasteurized milk, farm animals and house dust in early life protects against allergies and asthma^2–7^. At the skin surface, bacterial diversity is also important for homeostasis, skin barrier function and immunity^8,9^. In atopic dermatitis (AD), the primary cause of which is a skin barrier defect, the predominance of *Staphylococcus aureus* (*S. aureus*) over commensals causes infective eczema flares^10,11^. Preclinical and early-stage clinical trials have demonstrated that coagulase-negative staphylococci and other skin commensals attenuate eczema severity, opening the possibility of using bacterial products in the treatment of AD^12–19^. The exact mechanism of action is unclear but may involve anti-microbial peptides (AMPs) that suppress *S. aureus* growth^20–22^.

We have previously shown that second immunoglobulin-binding protein (Sbi) from the *S. aureus* secretome (FSA) is critical to inducing the release of constitutive interleukin (IL)-33 from human keratinocytes, thereby triggering AD^23^. We hypothesized that immunomodulatory factors released by the skin microbiome into a relatively nutrient-poor environment suppress the proinflammatory effects of *S. aureus* Sbi in AD by direct effects on keratinocytes. Here, we explored whether these bacterial immunomodulatory factors do indeed exist and delineate their chemical structure and mechanism of action. The long-term aim is to develop a novel therapy for treating allergic diseases.

In this study, we demonstrate that as nutrients become depleted, all staphylococcal species tested release lipopeptides from their cell walls that suppress type 2 immune responses by human keratinocytes in primary culture. Furthermore, when applied topically to the skin of NC/Tnd mice, diacylated lipopeptides suppress IL-33 release and the development of eczema in this mouse model. Their activity is completely abrogated by splitting the lipid from the peptide with lipoprotein lipase.

We expected the mechanism of action to involve interaction with the CD14/toll-like receptor (TLR) signaling pathway, but this was not the case as specific chemical inhibitors of TLR had no effect. In contrast, we observed that IL-33 release from the nucleus was suppressed, as after treatment with lipopeptides, this type 2 immune cytokine accumulated in the perinuclear space of keratinocytes. After activation by caspase, pore-forming gasdermin D (GSDMD) is known to promote the release of IL-33 and activity of this pathway was found to be linked to lipopeptide immunosuppressive activity.

The novel findings of this study suggest a class of novel, chemically stable, non-infectious chemicals that may be useful in treating not only allergic skin diseases but potentially respiratory and other allergic diseases^24^.

## Results

### Bacteria release factors that suppress type 2 immunity and eczema

**Fig. 1:**
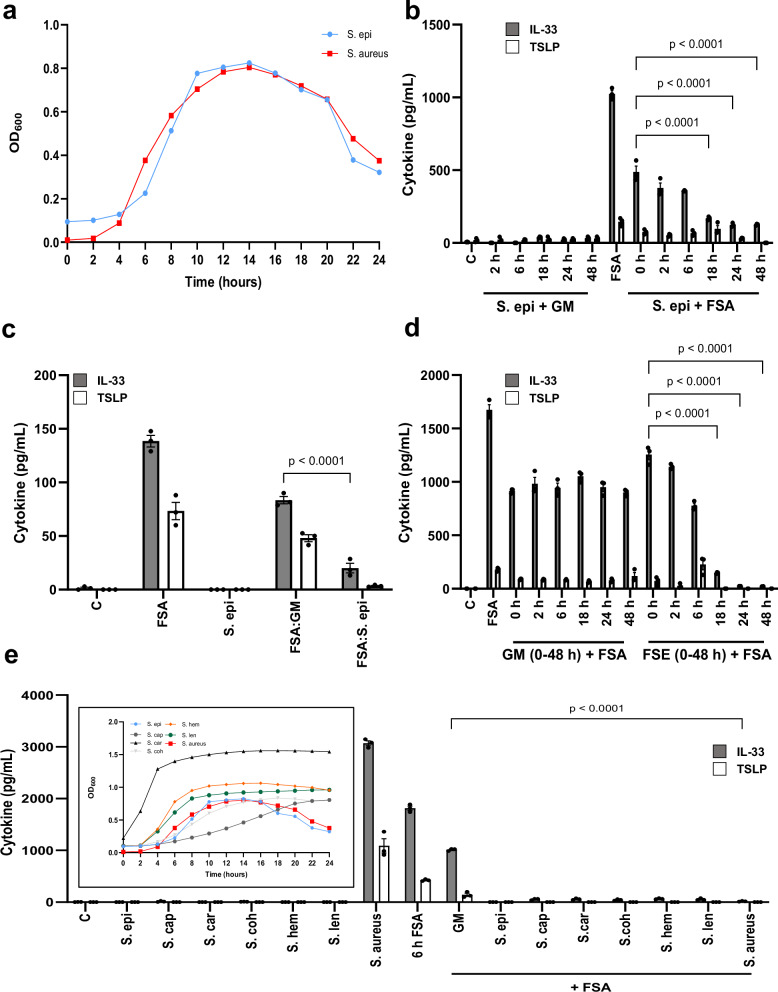
Bacteria in their stationary/death phase release soluble factors that abrogate IL-33 and TSLP release in vitro and ex vivo. **a** Growth curves of *S. aureus* (red line) and *S. epidermidis* (blue line) demonstrating logarithmic (4–8 h) and stationary/death (10–24 h) phases. **b** 10^7^ CFU/mL live *S. epidermidis* (S. epi) measured by the Miles & Misra method grown to ≥18 h inhibit *S. aureus* secretome (FSA)-induced IL-33 and TSLP release by NHEK after 6 h of co-culture. **c** Live *S. epidermidis* (S. epi) also suppresses FSA-induced IL-33 and TSLP release in tape-stripped human skin explants ex vivo. **d** The soluble *S. epidermidis* (FSE) inhibitory effect is only observed if extracted from 18 and 48 h bacterial culture. **e** Live *S. epidermidis*, *S. capitis, S. carnosus, S. cohnii, S. haemolyticus, S. lentus*, and *S. aureus* bacteria in their stationary phase at 18 h all completely suppress the type 2 immune activity induced by FSA. Insert demonstrates relative growth curves of these bacteria. Data are represented as mean ± SEM of three independent experiments performed in triplicate. *P*-values were determined by two-way ANOVA with Dunnett’s multiple comparisons test (**b**–**e**) relative to FSA:GM control. IL-33 and TSLP were measured by ELISA. FSA: *S. aureus* secretome from 6 h culture in keratinocyte growth media 2, FSE: 0−48 h *S. epidermidis* secretome in keratinocyte growth media 2, GM/C: keratinocyte growth media 2, S. aureus: live *S. aureus*, S. epi: live *S. epidermidis*, NHEK: normal human epidermal keratinocytes. Source data are provided as a Source data file.

In its logarithmic growth phase, *S. aureus* releases Sbi that triggers a type 2-immune response^23^. *S. epidermidis*, a common skin staphylococcal commensal, does not express Sbi and does not induce IL-33 release from keratinocytes despite a comparable growth rate (Fig. 1a, b). However, as nutrients become depleted and bacteria enter their stationary/death phases, *S. epidermidis* suppresses IL-33 and thymic stromal lymphopoietin (TSLP) secretion by *S. aureus* stimulated primary human epidermal keratinocytes (NHEK) in vitro (Fig. 1b). Similar inhibitory activity can be demonstrated ex vivo in tape-stripped human skin explants (Fig. 1c, Supplementary Fig. S1a). *S. epidermidis* blocks IL-33 and TSLP release even when separated from NHEK by a 0.4 μm polycarbonate filter, indicating that direct contact between bacteria and skin cells is not essential (Supplementary Fig. S1b). Immunomodulatory activity of the *S. epidermidis* secretome (FSE) increases in the post-logarithmic phase, suggesting an association with nutrient depletion (Fig. 1d). Other staphylococcal species, including *S. aureus*, release immunomodulatory factors after reaching their stationary growth phase (Fig. 1e), demonstrating that immunomodulatory activity is not unique to *S. epidermidis*. There is no evidence that bioactivity relates to cell death (Supplementary Fig. S1c). Staphylococcal species show similar dose response and EC50 for IL-33 inhibition, with the exception of *S. carnosus* which had a more exuberant growth curve (Fig. 1e insert) and was less bioactive at lower colony-forming units (CFUs) (Supplementary Fig. S2c).

**Fig. 2:**
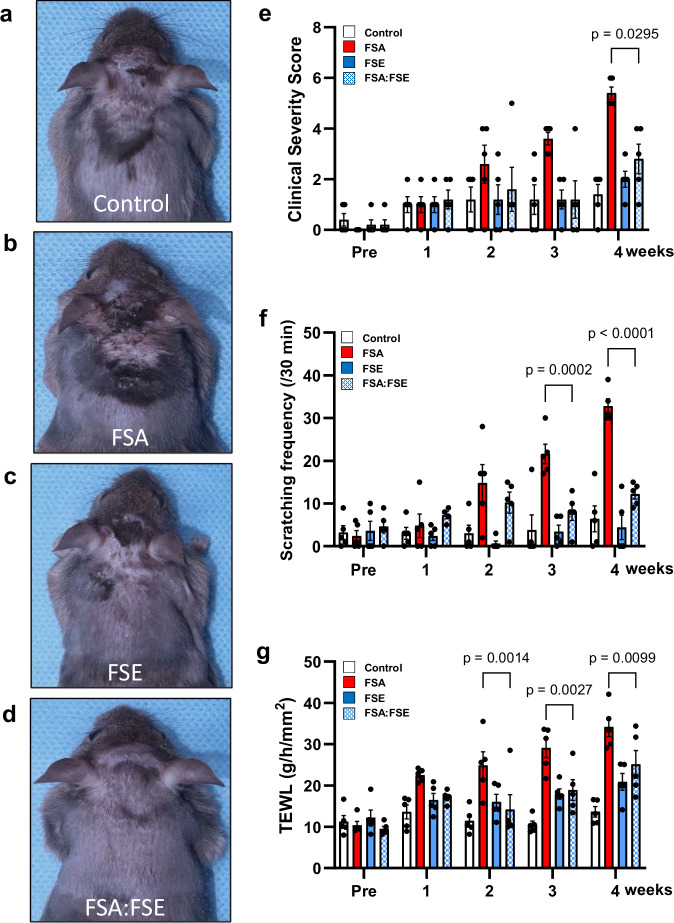
*S. epidermidis* secretome suppresses eczema in the NC/Tnd mouse model. **a**–**d** Representative images of NC/Tnd mice exposed to FSA for 4 weeks with or without daily topical FSE application. **e** Clinical severity score, **f** Scratching frequency, **g** Transepithelial water loss (TEWL). White bars: SDS topical treatment control, red bars: SDS topical treatment with *S. aureus* derived FSA, blue bars: SDS topical treatment with 18 h *S. epidermidis* derived FSE, hatched bars: SDS topical treatment with FSA and 18 h FSE. All data show results of five individual mice/group and mean ± SEM. Data were combined from three independent experiments. *P-*values were determined by two-way ANOVA with Tukey’s multiple comparisons test (**e**–**g**). Control: keratinocyte growth media 2, FSA: 6 h *S. aureus* Nm strain secretome, FSE: 18 h *S. epidermidis* secretome; TEWL: Transepithelial water loss. Source data are provided as a Source data file.

In vivo, 18 h FSE also suppressed the development of *S. aureus*-induced eczema in the NC/Tnd mouse model (Fig. 2a–g). Clinical scores (Fig. 2e) and scratching behavior (Fig. 2f) of mice were reduced, as was disruption to the skin barrier measured by transepithelial water loss (TEWL) (Fig. 2g).

### Bacterial immunomodulators are small, heat and protease stable molecules

To elucidate the chemical nature of these bacterial immunomodulatory factors, bioactivity of the FSE was tested after digestion with trypsin (TY) and heat treatment to 95 °C (HT). Neither treatment affected bioactivity, in keeping with the factor being non-proteinaceous (Supplementary Fig. S1d). Bioactivity from *S. epidermidis*, *S. carnosus*, *S. haemolyticus*, and *S. aureus* secretome all eluted in the same fractions near the total volume of a size exclusion Superose 12^®^ fast protein liquid chromatography (FPLC) column, suggesting common bioactive factor(s) of similar molecular weight (Supplementary Fig. S3).

### Bacterial immunomodulators are lipopeptides

**Fig. 3:**
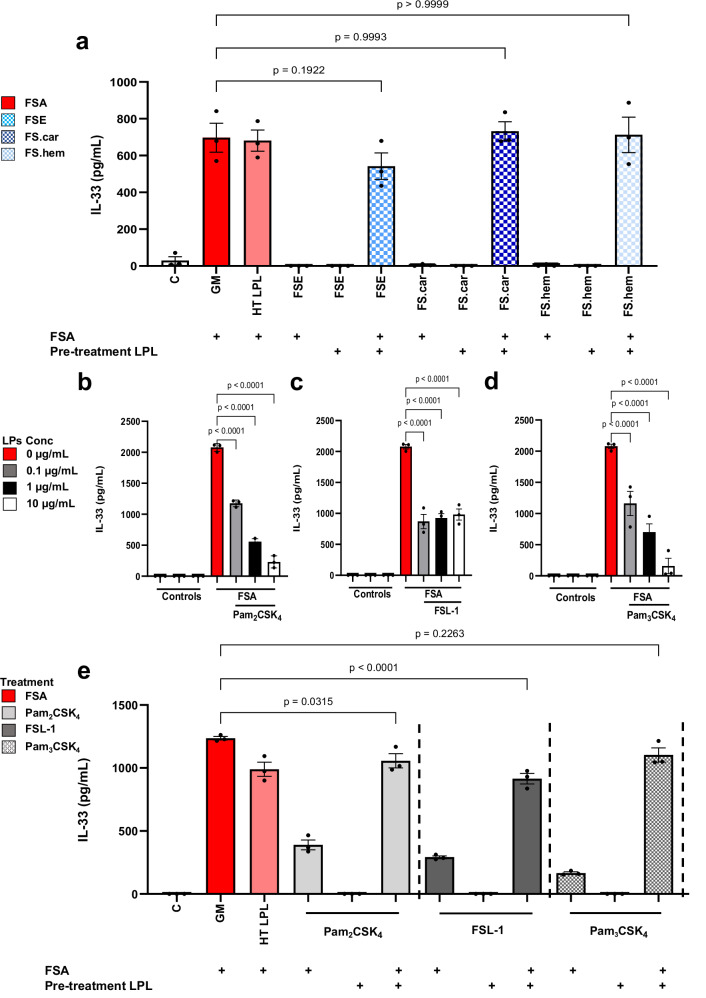
Immunomodulatory activity of staphylococcal secretome and synthetic lipopeptides are destroyed by lipoprotein lipase (LPL). **a** LPL (1200 U/mg:5 µg/mL) abrogates the inhibitory effect of *S. epidermidis*-derived 18 h FSE, and supernatants derived from other bacteria (*S. carnosus* (FS. car)*, S. haemolyticus* (FS. hem), in their post-logarithmic growth phase. Inhibitory effect on FSA-induced IL-33 released by NHEK of **b** Pam_2_CSK_4_ (0.1–10 µg/mL); **c** Pam_2_CGDPKHPKSF (FSL-1) (0.1–10 µg/mL); **d** Pam_3_CSK_4_ (0.1–10 µg/mL). **e** LPL (1200 U/mg) also abrogates the inhibitory effect of these synthetic lipopeptides: Pam_2_CSK_4_ (10 µg/mL), FSL-1 (10 µg/mL), and Pam_3_CSK_4_ (10 µg/mL). Data are represented as mean ± SEM of three independent experiments performed in triplicate. *P*-values were determined by one-way ANOVA with Dunnett’s multiple comparisons test (**a**, **e**) relative to FSA:GM control, Tukey’s multiple comparisons test (**b**–**d**). IL-33 was measured by ELISA. GM/C: keratinocyte growth media 2, heat-treated LPL: heat-treated lipoprotein lipase in GM, FSA: 6 h *S. aureus* secretome, FSE: 18 h *S. epidermidis* supernatant, LPL: Pseudomonas species lipoprotein lipase; Pam_2_CSK_4_ and Pam_2_CGDPKHPKSF (FSL-1): synthetic diacylated lipopeptides, Pam_3_CSK_4_: synthetic triacylated lipopeptide. Controls (**b**–**d**) represent IL-33 release from NHEK treated with GM, the lipopeptide, or the lipopeptide reconstitution vehicle only. Source data are provided as a Source data file.

In view of their low molecular weight, heat and TY stability, we explored the possibly that the immunomodulatory factors were bacterial wall lipopeptides released from the bacterial cell wall as nutrients become depleted in their stationary/death phases^25^. Lipoprotein lipase (LPL) is known to degrade mono-, di-, and triacylated lipopeptides, as well as the synthetic lipopeptide Pam_3_CSK_4_^26^. We show that pre-treatment of all bacterial stationary phase supernatants with LPL abolishes their immunomodulatory activity (Fig. 3a). To confirm the loss of acyl groups, we used MALDI-TOF MS/MS analysis before and after LPL treatment of the active fraction from the *S. epidermidis* secretome, as well as Pam_2_CSK_4_ and Pam_3_CSK_4_ (Supplementary Fig. S4). LPL treatment of Pam_2_CSK_4_ reduced the parent peak (1,271), resulting in fragments of 1033 (equivalent to loss of one fatty acid chain) and 672 (peptide only) (Supplementary Fig. S4a, b). Similarly, treatment of Pam_3_CSK_4_ (parent peak 1510) resulted in peaks corresponding to Pam_2_CSK_4_ after loss of each fatty acid moiety (1271, 1033, and 672) (Supplementary Fig. S4c, d). Mass spectroscopy analysis of *S. epidermidis* FPLC active fraction showed a similar breakdown after LPL treatment from a parent peak of 1329, creating dominant peaks of 656 and 582, in keeping with the loss of two or three acyl groups (Supplementary Fig. S4e, f). These data demonstrated the presence of acyl moieties within the immunomodulatory component of *S. epidermidis*, in keeping with di- or triacylated lipopeptides. Supporting this finding, synthetic di- (Pam_2_CSK_4_ and FSL-1) and tri- (Pam_3_CSK_4_) acylated lipopeptides inhibited FSA-induced IL-33 release from primary human keratinocytes (Fig. 3b–d). LPL treatment completely abolished their immunomodulatory activity (Fig. 3e).

We conducted additional experiments to determine which isomers and acyl structures were critical for immunosuppressive activity. We found that both *R*- and *S*-isomers of Pam_2_CSK_4_ were active (Supplementary Fig. S5a, b), while the nonacylated dehydrocorydaline alkaloid lipopeptide (Dhc-SK_4_) and monoacylated (Pam_1_CSK_4_) were not active (Supplementary Fig. S5c, d). Furthermore, one of the predicted active components of *S. epidermidis* by MALDI-TOF MS/MS was a lipopeptide with possible parent peptide sequence of CS_3_K. Synthetic Pam_2_CS_3_K was also active against both FSA and recombinant (r)Sbi (Supplementary Fig. S5e, f). Collectively, these data illustrate the structural diversity of these immune modulatory lipopeptides, whilst maintaining essential requirements for at least two acyl groups.

**Fig. 4:**
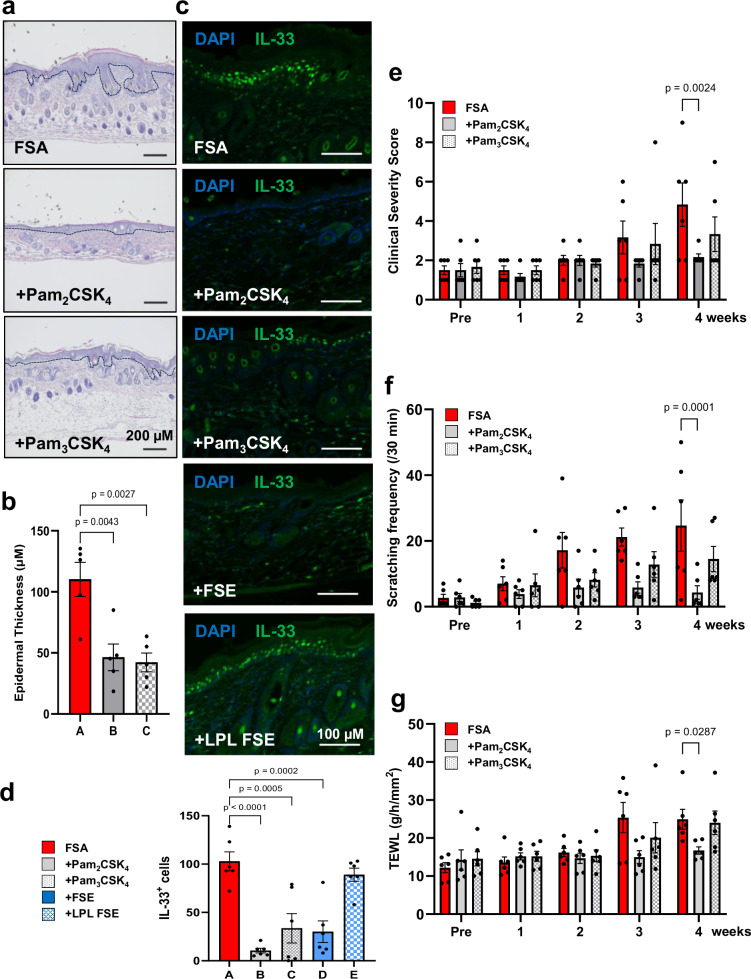
Synthetic bacterial lipopeptide Pam_2_CSK_4_ and to a lesser extent Pam_3_CSK_4_ suppress epidermal IL-33 expression and eczema in the NC/Tnd mouse model. NC/Tnd mice were exposed to *S. aureus* derived FSA for 4 weeks with or without daily application of topical Pam_2_CSK_4_ (10 µg/mL), or Pam_3_CSK_4_ (10 µg/mL). **a**, **b** Epithelial skin thickness and **c**, **d** IL-33 expression was assessed in skin biopsies taken at week 4. Red bars: SDS topical treatment with *S. aureus* derived FSA, gray bars: SDS topical treatment with Pam_2_CSK_4_, hatched bars: SDS topical treatment with Pam_3_CSK_4_, blue bars: SDS topical treatment with *S. epidermidis* derived FSE, blue hatched bars: SDS topical treatment with LPL treated *S. epidermidis* derived FSE. Mice were scored weekly for **e** clinical eczema severity, **f** scratching frequency, and **g** transepithelial water loss (TEWL). All data show results of six individual mice/group and mean ± SEM. Data combined from three independent experiments. *P-*values determined by one-way ANOVA with Tukey’s multiple comparisons test (**b**, **d**), two-way ANOVA with Tukey’s multiple comparisons test (**e**–**g**). Scale bars = 200 µM (**a**) and 100 µM (**b**). FSA: 6 h *S. aureus* Nm strain secretome, FSE: 18 h *S. epidermidis* secretome, Pam_2_CSK_4_: synthetic diacylated lipopeptide, Pam_3_CSK_4_: synthetic triacylated lipopeptide, TEWL: transepithelial water loss. Source data are provided as a Source data file.

We next investigated the in vivo effects of Pam_2_CSK_4_ and Pam_3_CSK_4_ on epidermal IL-33 expression and eczema severity when applied daily for four weeks to NC/Tnd mice housed in pathogen-free environments with or without FSA. In keeping with our in vitro results, both diacylated and triacylated lipopeptides normalized epidermal thickness (Fig. 4a, b). Pam_2_CSK_4_, Pam_3_CSK_4_ and FSE also reduced epidermal IL-33 expression when added with FSA, and FSE immunomodulatory activity was abrogated by LPL (Fig. 4c, d). Clinically, topical administration of Pam_2_CSK_4_ significantly suppressed eczema severity (Fig. 4e), reduced scratching behavior (Fig. 4f) and improving TEWL (Fig. 4g). Trends to less severe clinical eczema and scratching behavior with Pam_3_CSK_4_ did not reach statistical significance.

### Bacterial lipopeptides block IL-33 release from human keratinocytes

**Fig. 5:**
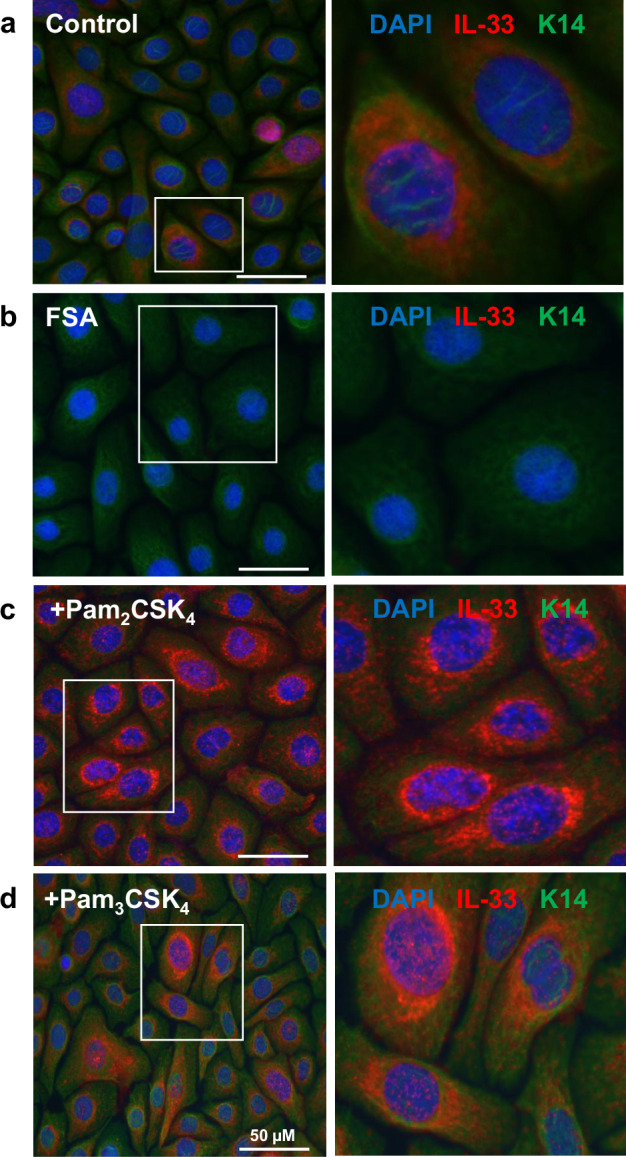
Lipopeptides prevent IL-33 release from human keratinocytes. Immunofluorescent staining of NHEK stimulated with **a** GM, **b** FSA, **c** FSA and Pam_2_CSK_4_ (10 µg/mL), or **d** FSA and Pam_3_CSK_4_ (10 µg/mL) for 6 h. Cells stained with anti-human IL-33 (red), anti-human cytokeratin 14 (green), and DAPI (blue). Similar results were observed from four independent experiments. Scale bar = 50 µM. FSA: 6 h *S. aureus* secretome, GM: keratinocyte growth media 2, Pam_2_CSK_4_: synthetic diacylated lipopeptide, Pam_3_CSK_4_: synthetic triacylated lipopeptide.

IL-33 are able to induce a type 2 immune response via passive release from dying cells, or in an active two-step process from living cells^27^. The first step in living cells involves the transition of IL-33 through nuclear membrane pores into the cytoplasm^28^. The second step requires transition of IL-33 through cell membrane pores into the extracellular space^29^. Using immunocytochemistry (ICC), we demonstrate that stimulation with FSA leads to release of constitutive IL-33 from the cell (Fig. 5b) compared to control (Fig. 5a). When Pam_2_CSK_4_ or Pam_3_CSK_4_ are added, IL-33 accumulates at the perinuclear rim (Fig. 5c, d), indicating inhibition of active release of IL-33 from the nucleus. There was no evidence of cell death by co-culture with either of these lipopeptides (Supplementary Fig. S1e, f).

### Immune modulation by lipopeptides is through caspase-GSDMD rather than TLR2 signaling

Standard dogma is that bacterial and synthetic lipopeptides induce a proinflammatory response by triggering TLR2/1 or TLR2/6 signaling. The toll/interleukin-1 receptor domain (TLR2 TIR) inhibitor TLR2-C29 did not suppress the immunomodulatory effect of FSE, Pam_2_CSK_4_ or Pam_3_CSK_4_ (Supplementary Fig. S6a–f). As only the R-isomer is known to activate TLR2^30^, our observation that both R- and S- Pam_2_CSK_4_ isomers are activity suggests a non-TLR mechanism of action. We therefore investigated alternative mechanisms of action.

Gasdermins are pore-forming molecules expressed by many cell types, particularly gut and skin epithelium^31^. After activation by caspases, GSDMD mediate pyroptosis and release of cytokines lacking signaling peptides such as IL-1β.^32^ In the lung, bacteria, fungi and house dust mite derived proteases can trigger GSDMD-mediated IL-33 release by pulmonary airway epithelium^29^. However, in our in vitro keratinocyte model, there was no evidence of cell death after FSA or rSbi stimulation of keratinocytes over 24 h (Supplementary Fig. S6g), or necroptosis, as the receptor-interacting protein 1 (RIP1) inhibitor necrostatin-1 had no effect (Supplementary Fig. S6h).

**Fig. 6:**
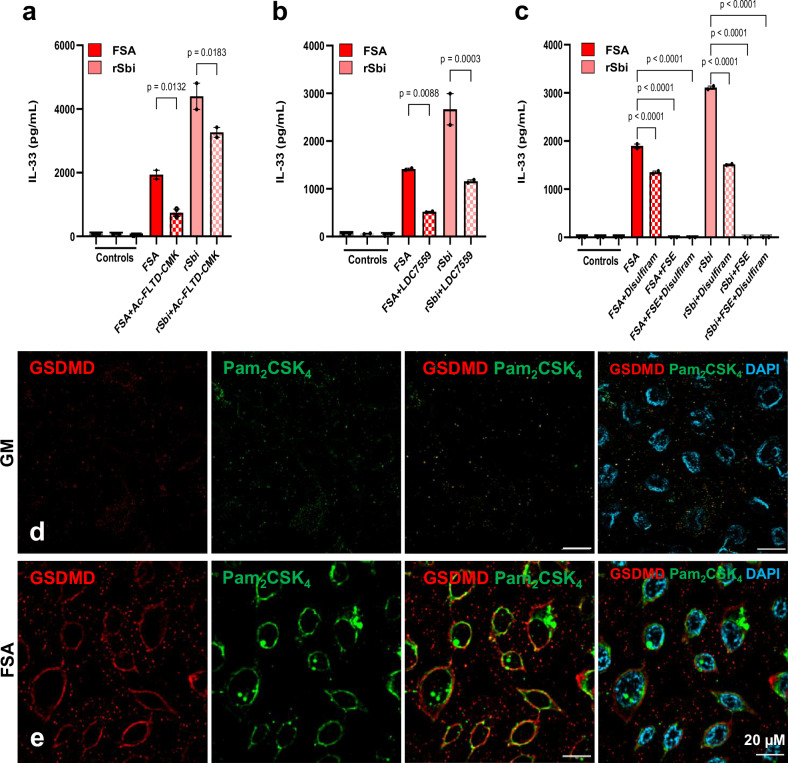
*S. epidermidis* derived- and synthetic lipopeptides suppress gasdermin D (GSDMD)-associated FSA and Sbi-induced IL-33 release from the nucleus by primary human keratinocytes. IL-33 secretion from NHEK induced by both FSA and rSbi are inhibited by the GSDMD inhibitors Ac-FLTD-CMK (10 µM) (**a**), LDC7559 (5 µM) (**b**), Disulfiram (1 µM), with or without FSE (**c**). Immunofluorescent staining of NHEK **d** at baseline (GM), and **e** stimulated with FSA and FITC Pam_2_CSK_4_ (10 µg/mL) for 10 min show colocalization of the diacylated lipopeptide and GSDMD. Cells were stained with anti-human GSDMD (red), Pam_2_CSK_4_ (green), and DAPI (blue). Data are represented as mean ± SEM of three independent experiments performed in triplicate (**a**–**c**), or in duplicate (**d**, **e**). *P*-values were determined by one -way ANOVA with Tukey’s multiple comparisons test (**a**–**c**). Scale bar = 20 μM. IL-33 was measured by ELISA. FSA 6 h *S. aureus* secretome; FSE 18 h *S. epidermidis* secretome; GM: keratinocyte growth media 2; Pam_2_CSK_4_: synthetic diacylated lipopeptide; rSbi: recombinant Sbi. Controls represent IL-33 release from NHEK treated with either GM, GSDMD inhibitor reconstitution vehicle, or the GSDMD inhibitor only. Source data are provided as a Source data file.

We therefore investigated whether FSA-triggered IL-33 release by keratinocytes might be secondary to sublethal caspase and subsequent GSDMD activation. Indeed, the pan-caspase inhibitor Z-VAD-FMK, and Ac-FLTD-CMK which specifically binds the active site of caspases 1, 5, and 4 preventing cleavage of GSDMD^33^, blocked FSA-induced IL-33 release from NHEK in a dose-dependent fashion (Supplementary Fig. S6i, Fig. 6a). Downstream inhibition of GSDMD pore formation using LDC7559 which binds directly to GSDMD preventing activation and subsequent insertion into the cell membrane^34^ and disulfiram which prevents the formation of GSDMD-NT pores^35^ also suppressed FSA and rSbi-induced IL-33 release by NHEK (Fig. 6b, c).

Using confocal microscopy with fluorescein isothiocyanate (FITC)-labelled Pam_2_CSK_4_ and 4’,6-diamindino-2-phenylindole (DAPI) to stain the nucleus, we clearly demonstrate that rather than being cell membrane bound, after stimulation with FSA the fluorescent lipopeptide is internalized and co-localizes with GSDMD in the perinuclear space of human keratinocytes (Fig. 6e). We conclude that lipopeptides suppress FSA and rSbi-induced release of IL-33 from live keratinocytes through a caspase/GSDMD-dependent signaling pathway.

## Discussion

Depletion of nutrients results in physiological and morphological changes in bacteria, slows growth and ultimately leads to their death^36^. Here we demonstrate that as nutrients become depleted and bacteria reach their stationary/death phase, low molecular weight lipopeptides are released from their cell walls that can suppress *S. aureus*-induced keratinocyte release of IL-33 and eczema in the NC/Tnd mouse model. The importance of IL-33 to our NC/Tnd mouse model was previously demonstrated by prevention of eczema by a neutralizing anti-IL-33 biologic^23^, and in this study by the reduction of epidermal IL-33 expression after topical application of lipopeptide. Immunomodulatory activity is not specific to one bacterial species but ubiquitous to all staphylococcal species we tested. Most staphylococci had a similar dose response. The exception was *S. carnosus*, a staphylococcal species not normally found on the skin. It had a different growth trajectory and therefore may have reached the stationary phase later. Furthermore, its lipopeptides are known to consist of very short-chain C2 fatty acid side chains, which may well help to explain the difference in their bioactivity^37^. The proinflammatory activity of Sbi produced by *S. aureus* can also be modulated by release of its lipopeptides as these bacteria reach their stationary phase, highlighting the capacity for self-immunomodulation of type 2 immune responses by this opportunistic pathogen. Enzymatic degradation with LPL that deacetylates lipopeptides^26^ completely abrogated the immunomodulation demonstrating that intact lipopeptides are essential for bioactivity. Di- and triacylated lipopeptides, but not monoacylated lipopeptides, were immunomodulatory in vitro. Interestingly, testing lipopeptides in our NC/Tnd eczema mouse model showed that diacylated lipopeptides were more bioactive. Triacylated lipopeptides, while reducing epidermal hyperplasia and to a less extent epithelial IL-33 expression, did not significantly improve eczema and scratching frequency. Differences in molecular size and hydrophobicity are likely to influence lipopeptide penetration of the stratum corneum and may explain these differences^38^. These results highlight the importance of animal and human trials when translating basic research findings into potential novel therapies.

Our findings are unexpected, as current dogma teaches that microbe-associated molecular patterns (MAMPs) such as bacteria-derived lipopeptides act via CD14/TLR signaling to stimulate host innate immunity^39,40^. Here we clearly show that in keratinocytes, not only are bacterial lipopeptides immunomodulatory rather than immunostimulatory, but the mechanism does not involve CD14 or TLR2 signaling. Furthermore, both *R*- and *S*-diacyl lipopeptide isomers of Pam_2_CSK_4_ were bioactive, supporting our conclusion that these molecules do not act via TLR2^30^.

Microbes, including *S. aureus*, can induce release of proinflammatory cytokines including IL-33 by sublethal caspase activation without inducing overt cell death^41,42^. In turn, caspases activate GSDMD that form pores in both nuclear and cell membranes through which cytokines pass into the extracellular milieu^31,43,44^. Although GSDMD-induced pore formation classically triggers pyroptosis (microbe-induced cell death), we show that in our experimental model, FSA and rSbi do not induce necrotic or pyroptotic keratinocyte cell death^23^. Others have also shown that allergen proteases can also induce non-classical activation of GSDMD and IL-33 release in the absence of cell death^29^. Here we demonstrate that FSA and Sbi-induced IL-33 release is suppressed by specific caspase and GSDMD inhibitors, that lipopeptides are taken up by keratinocytes and induce perinuclear pooling of IL-33. As GSDMD induced pore formation occurs not only in the outer cell membrane but also the nuclear membrane^41,42^, its colocalization with the lipopeptides in the perinuclear rim suggests that inhibition of GSDMD and nuclear membrane pore formation is the reason for observed lack of IL-33 release.

Our study provides an alternative view of bacterial lipopeptides that contrasts with the classical dogma of immune stimulation through TLR pathways. In the crosstalk between host and microbe at the skin barrier, we show that bacteria in their stationary phase ubiquitously shed lipopeptides as a biproduct of cell wall turnover. In keratinocytes, these soluble bacterial cell wall components act not as danger signals, but rather to promote host surface epithelium homeostasis. Our data suggests that there is no single immunomodulatory lipopeptide structure, but rather a functional convergence on di- and triacyl molecules. Further work is required to link these Gram-positive di- and triacyl lipopeptides to previous epidemiological observations that people exposed to bacterial products in unpasteurized milk and barns of traditional farms suffer less allergic hypersensitivity and disease^45,46^. Farm dust has been found to reduce IL-33 expression in bronchial epithelial cells and it will be interesting to determine if bacterial lipopeptides might also modulate the immune homeostasis in the lungs^47^.

Scientists and physicians search for an elixir to protect against allergies. The findings presented in this study illustrate the intricacies of host-microbe interaction, where bacterial commensals can function as immune modulators depending on their environment. Undoubtedly bacteria have evolved to contribute to immune homeostasis. This study suggests that soluble bacterial lipopeptides offer chemically stable therapeutics for the treatment of AD and other inflammatory diseases. The challenge is now to conduct further studies using these lipopeptides in humans with AD and other atopic disorders to determine if they can be harnessed as therapeutics in clinical practice.

## Methods

### Study design

#### Research ethics

Our research complies with relevant ethical regulations. Human skin specimens sampling complied with the Manchester Skin Health Biobank, Research Ethics Committee number 24/NW/0044 with written consent obtained from all patients in accordance with the Declaration of Helsinki. All animal experiments complied with guidelines of University Animal Care and Use Committee of the Tokyo University of Agriculture and Technology and of Science Council of Japan for the use of laboratory animals.

#### Research objectives

The aim of the study was to determine the chemical nature and mechanism of action of immunomodulatory factors produced by skin commensals that suppress type 2 immune responses induced by *S. aureus*. Secondary aims were to test these purified bioactive compounds in an animal model of eczema to provide preliminary evidence for use in designing subsequent clinical trials in humans as potential therapy for allergic skin diseases, particularly AD.

#### Research cell cultures and animals

In vitro testing was performed using primary NHEK cells, ex vivo testing used human primary skin explants. Human in vitro and ex vivo results were corroborated in vivo using the NC/Tnd eczema mouse model.

#### Experimental design

An IL-33 ELISA readout from an in vitro human keratinocyte cell line model was used to assess the proinflammatory and immunomodulatory effects of soluble bacterial products from skin microbiota (a full list of abbreviations specific to this study are provided in Supplementary Table S1). The most active products were then purified by size-exclusion FPLC and their chemical structure elucidated by enzymatic digestion and MALDI-TOF mass spectroscopy. Immune mechanism of action was determined using specific inhibitors of cell signaling and cell death pathways alongside ICC. Confirmation of their clinical effect was determined using an in vivo NC/Tnd eczema mouse model. No relevant data has been excluded.

### Bacterial species and strains

Bacterial strains used in this study are listed in Supplementary Table S2. Methicillin-sensitive *S. aureus* (clinical strain, isolated from a chronic leg wound), and Newman (Nm) strain *S. aureus* were provided by Professor McBain, University of Manchester, UK and Professor Geoghegan, University of Birmingham, UK, respectively. Other staphylococcal species including *S. epidermidis, S*. *capitis, S. carnosus, S. cohnii, S. haemolyticus*, and *S*. *lentus*, were gifts from Dr Xia, University of Manchester, UK. Staphylococcal species and Nm were plated overnight (37 °C) on nutrient agar or tryptic soya agar, respectively, and quantified by Miles and Misra.

### Bacterial growth curves by optical density (OD) measurements

Overnight cultures of live bacteria were diluted to 1 × 10^5^ CFU/mL, or for dose response experiments, 10^3^–10^8^ CFU/mL, and bacterial growth/well was monitored over a 24 h period, at 2 h intervals by measuring the OD_600nm_ using the BioTek LogPhase 600 Microbiology reader and Gen5 software (BioTek), in a 96-well flat bottom plate, incubated at 37 °C.

### Preparation of filtered supernatants

A single bacterial colony/species was inoculated with 10 mL nutrient broth (or tryptic soya broth for Nm) overnight (37 °C). Cultures were washed twice with Dulbecco’s phosphate-buffered saline (PBS) and centrifuged (1600 × *g*, 5 min, room temperature (RT)). CFU/mL was adjusted by OD_600nm_ to 10^7^ CFU/mL, inoculated in 40 mL of Human Keratinocyte Growth Medium 2 (PromoCell) and incubated for 6–48 h at 37 °C in a shaking incubator (200 rpm). After incubation, samples were centrifuged (1600 *g*, 5 min, RT), supernatants collected, and filter sterilized using 0.2 µM filters. The filtered supernatants were then treated with 2% penicillin/streptomycin (Sigma-Aldrich®) and stored at −80 °C until required for stimulation experiments.

### Primary human keratinocyte culture and stimulation

Primary cells and reagents used in this study are listed in Supplementary Tables S2, S3 and S4. NHEK were cultured in Keratinocyte Growth Medium 2 (GM) plus supplements at 37 °C under 5% CO_2_ without antibiotics until 70–80% confluent. Cells were washed using (4-(2-hydroxyethyl)-1-piperazineethanesulfonic acid) (HEPES) Buffered Saline Solution and detached with Trypsin/EDTA (0.04%) for 5 min at 37 °C and subsequent addition of trypsin neutralizing solution. Following centrifugation (3 min, 220 × *g*), cells were seeded in 24-well tissue culture plates at a density of 5 × 10^4^ cells/well. Cells of passages 3–5 were used for experiments.

NHEK were stimulated with live *S. aureus* or 6 h FSA, and co-cultured with live, or 6 h and/or 18 h filtered supernatant from staphylococcal species 10^7^ CFU/mL for 6 h at 37 °C. Supernatants were collected after centrifugation (10,000 × *g*, 15 min, RT). For lipopeptide experiments, lipoprotein lipase *Pseudomonas sp*. (LPL) was used at a working concentration of >1200 U/mg (5 µg/mL) diluted in 18 h FSE for 30 min at 37 °C, then inactivated at 95 °C for 5 min, before a 6 h stimulation of NHEK with 1:1 ratio of 6 h FSA. Synthetic lipopeptides were reconstituted as per manufacturer instructions, filter sterilized and diluted in 6 h FSA to a working concentration of 0.1, 1, and 10 μg/mL, then incubated with NHEK for 6 h at 37 °C. For TLR inhibition experiments, the TLR2/4 antagonist TLR2-C29 was reconstituted according to manufacturer recommendations, filter sterilized and diluted to a working concentration of 0.6, 6, and 60 µg/mL in GM, then incubated with NHEK for 1 h at 37 °C, prior to co-treatment with 10^7^ CFU/mL 6 h FSA or rSbi (1 µg/mL):18 h FSE for 6 h at 37 °C and supernatants were collected as above. For GSDMD inhibition experiments, the inhibitors, Ac-FLTD-CMK, LDC7559 and disulfiram were used at 1 µM, 5 µM, and 10 µM, respectively, 30 min at 37 °C, before a 6 h stimulation of NHEK with rSbi (1 µg/mL).

### Transwell assay

Transwell assays were performed in 24-well transwell (6.5 mm diameter, 0.4 µM pore size, Costar®). 1 × 10^5^ NHEK/well were added to the lower chamber co-cultured with either live *S. aureus* or 6 h FSA. Live *S. epidermidis* (S. epi) or 18 h FSE was added to the upper chamber. After 6 h incubation at 37 °C, the supernatant was collected for analysis of type 2 cytokines by ELISA.

### Ex vivo skin organ culture

Human skin specimens were obtained from adult healthy patients following breast or abdominal reduction surgery or liposculpture procedures (Manchester Skin Health Biobank, Research Ethics Committee number 24/NW/0044), and all patients gave written informed consent according to the Declaration of Helsinki. Post-excision, subcutaneous fat tissue was removed, biopsies were taken using sterile 4 mm biopsy punches (KAI Medical, GP Supplies Ltd) and placed in 1 mL/biopsy William’s E medium (Thermo Fisher Scientific) supplemented with l-glutamine (2 mM, Sigma-Aldrich®), penicillin (100 U/mL)-streptomycin (0.1 mg/mL) (Sigma-Aldrich®), 0.02% (v/v) hydrocortisone (Sigma-Aldrich®) and 0.1% (v/v) insulin (Sigma-Aldrich®) in 6-well culture plates containing 0.4 µM ThinCert™ cell culture inserts (Greiner Bio-One) dermal side down. Intact epidermal exposed biopsies were tape stripped (ten consecutive tape strips for 10 s) using sterile adhesive stripping discs (3.8 cm^2^ D-Squame®) to remove the stratum corneum, and/or treated with 5 µL/biopsy of 6 h FSA and 10^7^ CFU/mL 18 h S. epi (live *S. epidermidis*) for 6 h (37 °C, 5% CO_2_). Following treatments, supernatants were removed and immediately stored at −80 °C. Skin biopsies were mounted in CellPath optimal cutting temperature (OCT) embedding matrix (Fisher Scientific), snap frozen in liquid nitrogen and stored at −80 °C. 5 µM sections were cut and stained with hematoxylin and eosin (H&E) to confirm removal of the stratum corneum.

### Human IL-33 and TSLP ELISA

R&D System ELISA were used to measure IL-33 (DY3625-05) and TSLP (DY1398-05) release from stimulated NHEK according to the manufacturer’s instructions. Optical densities were measured at 450 nm with a 540 nm reference wavelength, and IL-33/TSLP release was determined through comparison to a standard curve (VERSAmax™, Molecular Devices) using SoftMax software.

Briefly, high-binding 96-well plates were coated overnight with 50 μL/well of 0.8 µg/mL Capture antibody. The plate was then blocked with 150 μL/well 1X Reagent Diluent before addition of 50 μL/well of standard (2-fold serial dilution from 1500 pg/mL/2000 pg/mL, respectively) and unknown sample for 1 h at RT. Plates were further incubated for 1 h at RT with 50 μL/well of 50 ng/mL Detection antibody, followed by incubation with 50 μL/well of 1:40 dilution of Streptavidin-HRP for 20 min at RT. 50 μL/well 3,3'5,5’-tetramethylbenzidine (TMB) substrate (Sigma-Aldrich®) was then added for 20 min at RT before the final addition of 25 μL well Stop Solution (H_2_SO_4_).

### Lactate dehydrogenase (LDH) assay

LDH-Cytotoxicity Assay Kit II (Ab65393) was used to measure cytotoxicity in NHEK samples following specified treatments according to manufacturer’s instructions. Briefly, 10 µL NHEK conditioned medium was transferred to a 96-well plate with 100 µL of LDH reaction mix and incubated at RT in the dark for 30 min. OD at 450 nm was then measured (VERSAmax™, Molecular Devices) and LDH was calculated against low (NHEK + GM) and high (lysed NHEK) controls using SoftMax software.

### NC/Tnd mice

NC/Tnd mice are one of substrains of the NC/Nga mice maintained at Tokyo University of Agriculture and Technology. This mouse served as the original strain for NC/NgaTndCrlj mice distributed by Jackson Laboratories and is widely used for drug discovery and other applications as a spontaneous model mouse for atopic dermatitis. NC/Tnd mice were maintained in the laboratory of Comparative Animal Medicine at the Tokyo University of Agriculture and Technology. In the set of experiments described here, they were kept in specific-pathogen-free housing to prevent the natural development of eczema. The animal room was maintained at 22 ± 4 °C with a relative humidity of 40 ± 15% under a 12:12 h light–dark cycle, with ad libitum access to food and water. All animal experiments complied with the guidelines of University Animal Care and Use Committee of the Tokyo University of Agriculture and Technology (No. R05-98, R06-19, R07-38), as well as with the guidelines of Science Council of Japan for the use of laboratory animals. Only male mice were used in this study to reduce biological variability.

### Topical application of reagents to NC/Tnd mice

100 µL/mouse of treatment (6 h FSA and/or 18 h FSE/ LPL > 1200 U/mg (5 µg/mL) treated FSE) at a concentration of 10^8^ CFU/mL, Pam_2_CSK_4_ and Pam_3_CSK_4_ (all at 10 µg/mL) was topically applied to the back skin of male 6–7-week-old NC/Tnd mice, daily for four weeks. Skin barrier disruption was performed by 4% SDS (150 µL/mouse) 1 h before treatment. The number of animals used in each experiment is indicated in the corresponding figure legends.

### Clinical eczema severity and scratching behavior scoring of mice

Clinical eczema scores in mice were assessed as previously described^23^. Briefly, the total clinical severity score was defined in individual mice as the sum of the individual scores graded as 0 (none), 1 (mild), 2 (moderate), 3 (severe) for each of five signs and symptoms (itch, erythema/hemorrhage, oedema, excoriation/erosion, and scaling/dryness). Scratching frequency and duration were measured for 30 min each week and analyzed automatically using SCLABA^®^-Real system (Noveltec). All mice were kept in an acrylic cage for 30 min for acclimation before each measurement.

### Transepithelial water loss measurement in NC/Tnd mice

Transepithelial water loss (TEWL) was measured using Tewameter^®^ TM300 from Courage + Khazaka electronic GmbH, once weekly for four weeks. Temperature and humidity were maintained at 22 ± 0.5 °C and 50 ± 10%, respectively, before the measurements. The measurements for each mouse were taken 3 times, and the mean value was calculated.

### Epidermal skin thickness measurements in NC/Tnd mice

The thickness of the epidermis was measured from skin biopsies fixed and then stained with H&E. The formula epidermal thickness (μM) = epidermal area (μΜ^2^)/epidermal length (μM)^48^.

### IL-33 immunofluorescence staining of NC/Tnd mice

Paraffin-embedded skin sections from NC/Tnd mice (4 µM) were deparaffinized and subjected to antigen retrieval in Tris-EDTA buffer (10 mM Tris, 1 mM EDTA, pH 9.0) at 60 °C for 16 h, followed by natural cooling. After washing with wash buffer (1x TBS containing 0.1% Tween-20) and blocking with Block ACE solution (Bio-Rad), sections were incubated overnight at 4 °C with rabbit anti-mouse IL-33 (1:2000) diluted in antibody dilution buffer (25% Block ACE in wash buffer). Alexa Fluor™ 488-conjugated goat anti-rabbit IgG (1:1000, diluted in antibody dilution buffer) was applied for 1 h at RT after washing. Nuclei were counterstained with DAPI (1 µg/mL in 0.1% TBS-T). Sections were mounted with ProLong™ Gold and visualized using the BZ-X800 microscope (Keyence) under a high-powered field (HPF) at 200X magnification. Quantification was performed by counting IL-33-positive cells per HPF using ImageJ (Fiji v2.9.0).

### Fractionation of bacterial supernatant by chromatography

6 h and 18 h filtered supernatant from staphylococcal species were fractionated using size exclusion centrifugal filter columns (Amicon Ultra 15, 3-KDa cut-off membranes) and centrifuged for 5–7 min at 3000 × *g*, at 4 °C. Retained fractions were fractionated further using Superose^®^ 12 Fast-Protein Liquid Chromatography (FPLC) column (1–300 kDa) generating 52 fractions in phosphate-buffered saline (PBS). Activity was tested using an NHEK and FSA (filtered supernatant from *S. aureus*) co-culture system and tested for subsequent cytokine analysis, and mass spectrometry.

### MALDI-TOF mass spectrometry

10 µL (1 mg/mL purified and fractionated 18 h keratinocyte growth media and staphylococcal species) sample mixed with 10 µL matrix solution (10 mg/mL α-Cyano-4-hydroxycinnamic acid (Merck Group) in 50% ACN 0.1% TFA). 1.5 µL of this mixture was added to the center of a well on a polished steel target plate (Bruker Corp) and left to air dry until fully crystallized. This plate was loaded into a rapifleX mass spectrometer (Bruker Corp) operating in Reflectron Positive Mode <3 kDa mass range previously calibrated on PEG solution prepared similarly. FlexControl software (Bruker Corp) was used to optimize the laser settings for maximum resolution and sensitivity before acquiring 10,000 shots over 1 s using random partial well sample carrier setting (200, 2000 μM) and summed to produce spectra analyzed with flexAnalysis software (Bruker Corp). An Agilent (Agilent Technologies Inc) 1290 Infinity II HPLC was used to inject 5 µL of sample into 50% ACN (0.1% formic acid) mobile phase (Merck Group), which was introduced into a previously tuned and calibrated with reference Solution, Agilent (Agilent Technologies Inc) 6560 IMS QTOF mass spectrometer operating in either Positive or Negative mode, QTOF only. The resulting flow injection peak at RT 0.3 min was extracted using Agilent MassHunter Qualitative 10 software to produce the spectra.

### Immunocytochemistry (ICC)

1 × 10^4^ NHEK seeded on sterile cover slips in 24-well tissue culture plates until 90% confluent were treated with either GM, 6 h FSA, 18 h FSE and/or the TLR2 ligands Pam_2_CSK_4_ (10 μg/mL), Pam_2_CSK_4_ (Fluorescein-Aca-Aca) (10 μg/mL), and Pam_3_CSK_4_ (10 μg/mL) for 10 min or 6 h at 37 °C. Cells were fixed with 4% paraformaldehyde (20 min, RT), permeabilized with 1:100 dilution of Triton X-100 in PBS (15 min, RT) and blocked with either Avidin/Biotin (Vector Laboratories) or normal horse and goat serum (Vector Laboratories) (30 min, RT). NHEK were then incubated overnight at 4 °C with either mouse anti-human IL-33, rabbit anti-human GSDMD or anti-cytokeratin-14 polyclonal antibodies, followed by a secondary biotinylated mouse IgG antibody (40 min, RT) and Streptavidin Cy3 (40 min, RT) or goat anti-rabbit IgG conjugated with AlexaFluor® 488, or goat anti-rabbit IgG conjugated Texas Red (1 h, RT), followed by ProLong™ Diamond antifade mountant with DAPI. Stained cells were visualized using the Eclipse C*i*-L upright microscope (Nikon, M568E) and images acquired with the DS-F*i*3 microscope camera (Nikon, M667E) under DAPI, Cy3 and FITC filters. Images of individual channels were merged, and scale bars added using ImageJ (Fiji v2.9.0). For confocal microscopy, images were acquired on a ZEISS Lattice SIM 3 using Zen Blue software with a 40X/1.4 NA oil immersion objective and Hamamatsu ORCA-Fusion BT camera. Images were captured in SIM-apotome mode, with a 26.8 μM grating and 5 phases. Z-stacks were captured at intervals of 0.1 μM using the following exposure times and laser powers—DAPI [25 ms at 50% 405 nM]; Alexa488 [75 ms at 20% 488 nM]; Alexa 555 [75 ms at 50% 561 nM]. Processing was performed using SIM2 in 3D mode with Wiener filter order recombination, a sharpness filter of 10.5746, no regularization, 8 iterations, with X4 processing and X4 output sampling. Maximum intensity projections were created using Fiji/Image J.

### Statistical analysis

All in vitro and ex vivo experiments were carried out with a minimum of two to three technical and three biological replicates as detailed in the figure legends. In vivo experiments were performed three times with five to six mice per group. Normality of all data was tested using the Shapiro-Wilk test. If normality of data could be assumed, statistical significance of differences between groups were calculated using either one-way or two-way ANOVA followed by Tukey’s multiple comparisons or Dunnett’s post-hoc test. If normality could not be assumed using the QQ residual plot, statistical comparisons between groups were determined using the Kruskal–Wallis test with Dunn’s multiple comparisons and adjusted *P*-value reported. All analyses were performed using GraphPad Prism 10 Version 10.9.1.1 (GraphPad Software, Inc., CA, USA).

### Reporting summary

Further information on research design is available in the Nature Portfolio Reporting Summary linked to this article.

## Supplementary information

Supplementary Information

Reporting summary

Transparent Peer Review file

## Source data

Source Data File

## Data Availability

The main data supporting the findings of this study are available in the article or the Supplementary Source Data File. The mass spectrometry proteomics data have been deposited with the dataset identifier: Source Data File, Fig. S4 and in Zenodo: 10.5281/zenodo.18848797. Source data are provided with this paper.
