# Supplementary material for: Soluble bacterial lipopeptides suppress gasdermin D-associated IL-33 release in keratinocytes and atopic dermatitis in mice
Source: Nat Commun. 2026 May 13;17:6367. doi: 10.1038/s41467-026-72376-x (PMC13377172; doi:10.1038/s41467-026-72376-x)
Supplement: Supplementary file 1 — Supplementary Information [file 41467_2026_72376_MOESM1_ESM.pdf]

Supplementary Information

Soluble bacterial lipopeptides suppress gasdermin D-associated IL-33 release in keratinocytes and atopic dermatitis in mice

Williams, H., & Muko, R., et al.

*Corresponding author: peter.arkwright@manchester.ac.uk

Contents

Supplementary Figures: S1-S6

Supplementary Tables: S1-S4

Provided separately as a Supplementary Data File

Source Data File: The source data for Figures 1-6 and S1-S6

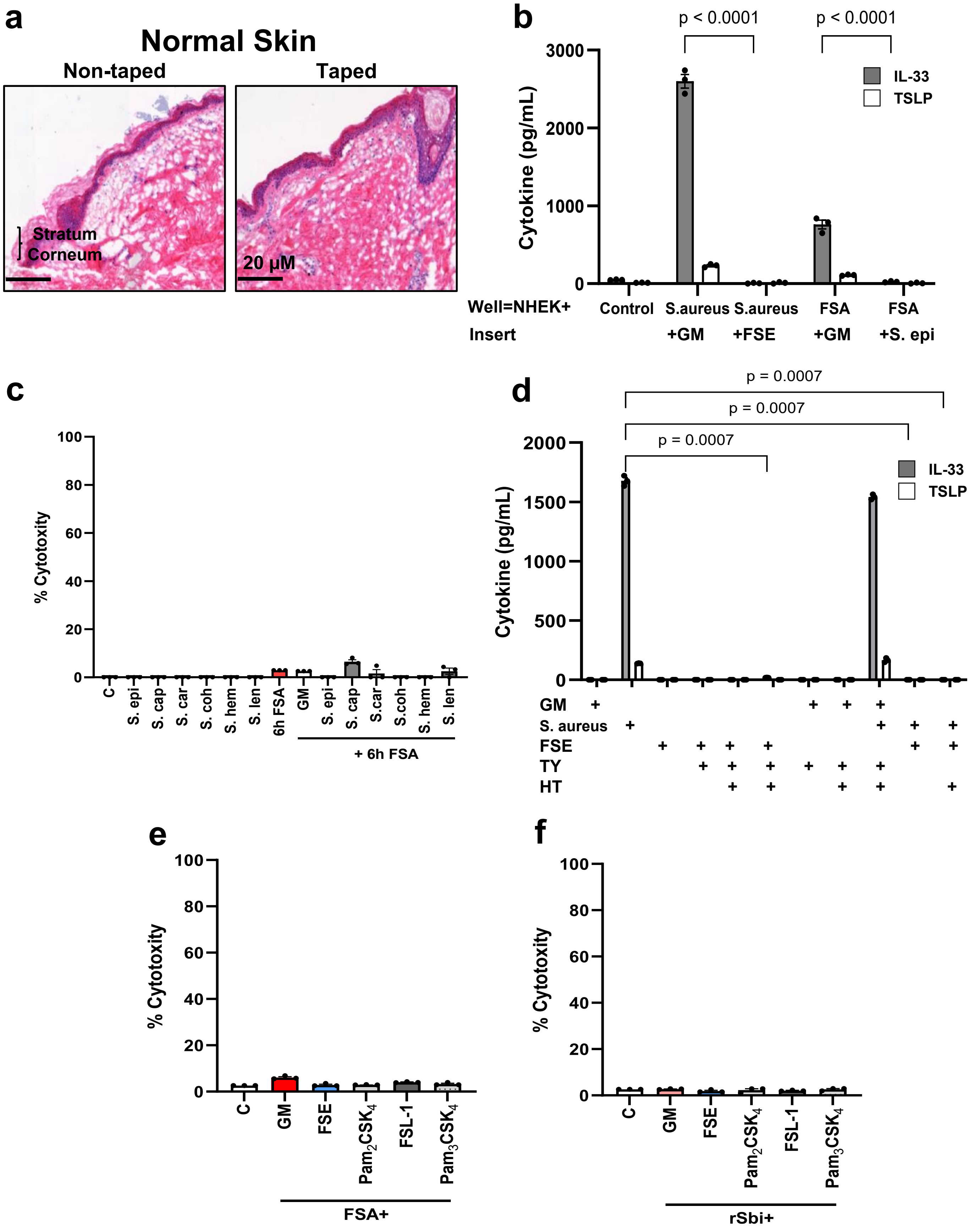

a
Normal Skin
Non-taped
Taped
Stratum Corneum
20 μM
b
p < 0.0001
p < 0.0001
Cytokine (pg/mL)
IL-33
TSLP
Well=NHEK+
Insert
Control
S.aureus +GM
S.aureus +FSE
FSA +GM
FSA +S. epi
c
% Cytotoxity
C
S. epi
S. cap
S. car
S. coh
S. hem
S. len
6h FSA
GM
S. epi
S. cap
S.car
S.coh
S. hem
S. len
+ 6h FSA
d
p = 0.0007
p = 0.0007
p = 0.0007
Cytokine (pg/mL)
IL-33
TSLP
GM
S. aureus
FSE
TY
HT
e
% Cytotoxity
C
GM
FSE
Pam2CSK4
FSL-1
Pam3CSK4
FSA+
f
% Cytotoxity
C
GM
FSE
Pam2CSK4
FSL-1
Pam3CSK4
rSbi+

Figure S1: Immunomodulatory activity of stationary phase bacteria is heat and protease stable and is not cytotoxic.

(a) H&E-stained ex vivo of non-taped versus taped normal skin sections confirming removal of the stratum corneum. Inhibitory activity derived from *S. epidermidis* (b) passes through a 0.4 μM polycarbonate transwell membrane. (c) Inhibition of FSA-induced IL-33 release from NHEK by staphylococcal commensal bacterial lipopeptides is not associated with cell death (no observable LDH activity). (d) Inhibitory activity derived from *S. epidermidis* is resistant to trypsin (TY) and heating to 95°C (HT). FSA (e) and rSbi (f) induced IL-33 release from NHEK by bacterial lipopeptides including FSE, Pam_2CSK_4 (10 μg/mL), Pam_2CGDPKHPKSF (FSL-1) (10 μg/mL), Pam_3CSK_4 (10 μg/mL) are not associated with cell death. Data are represented as mean ± SEM of three independent experiments performed in triplicate. P-values were determined by two-way ANOVA with Tukey's multiple comparisons test (b, d), Kruskal-Wallis with Dunn's multiple comparisons test (c, $P > 0.999$), one-way ANOVA with Dunnett's multiple comparisons test (e, f, ns: not significant). Scale bar = 200 μM. *S. epidermidis*, *S. capitis, S. carnosus, S. cohnii, S. haemolyticus and S. lentus.* FSE: 18 h *S. epidermidis* supernatant, Pam_2CSK_4 and Pam_2CGDPKHPKSF (FSL-1): synthetic diacylated lipopeptides, Pam_3CSK_4: synthetic triacylated lipopeptide. Source data are provided as a Source Data File.

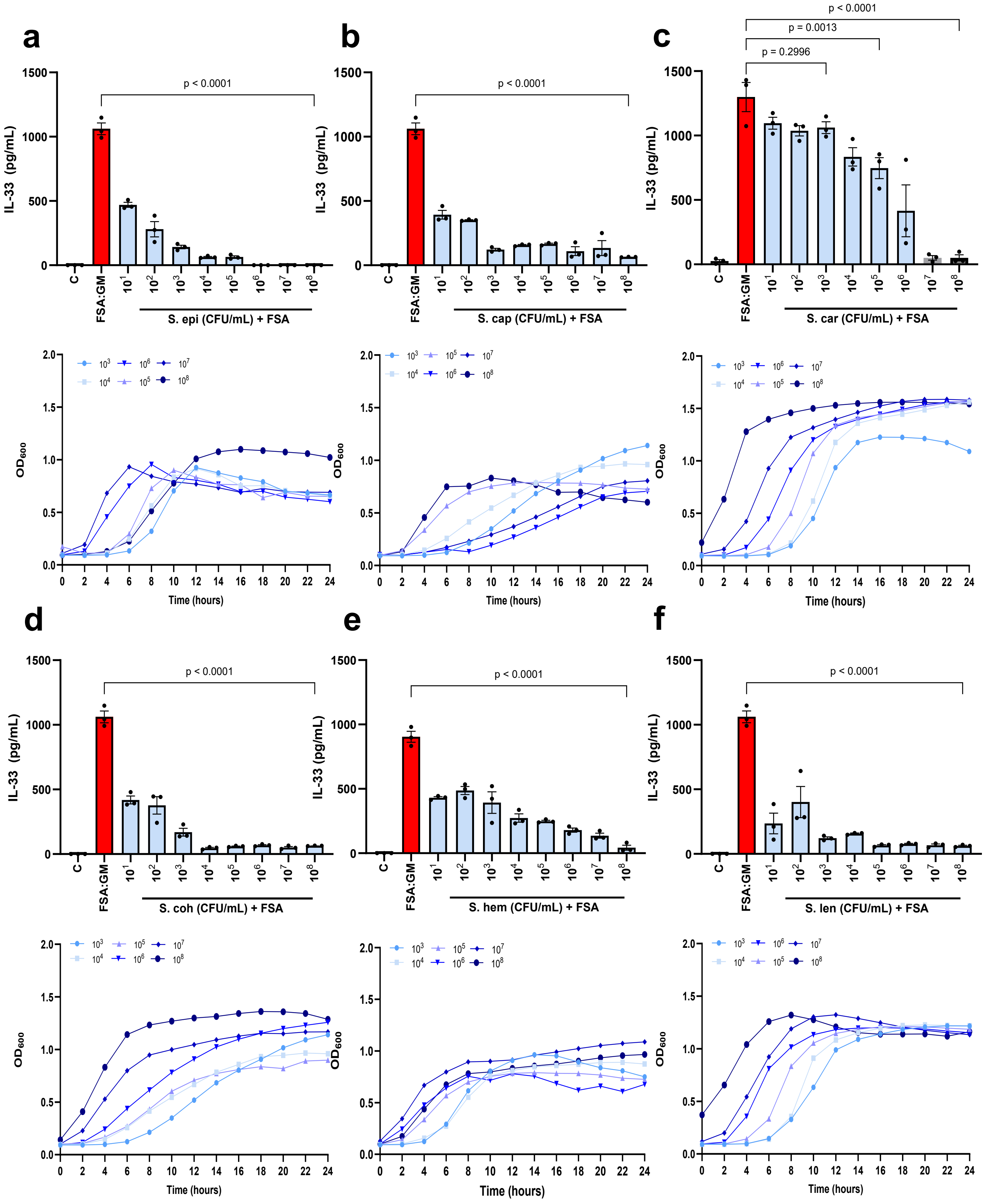

a
b
c
d
e
f
IL-33 (pg/mL)
p < 0.0001
p = 0.2996
p = 0.0013
C
FSA:GM
S. epi (CFU/mL) + FSA
S. cap (CFU/mL) + FSA
S. car (CFU/mL) + FSA
S. coh (CFU/mL) + FSA
S. hem (CFU/mL) + FSA
S. len (CFU/mL) + FSA
OD_{600}
Time (hours)

Figure S2: Dose response immunomodulation and growth curves of different staphylococcal species.

Bar graphs show dose response inhibition of FSA-induced IL-33 release by NHEK after addition of 18 h live bacteria with different starting CFU/mL, (a) *S. epidermidis*, (b) *S. capitis,* (c) *S. carnosus,* (d) *S. cohnii,* (e) *S. haemolyticus,* and (f) *S. lentus*. Line graphs represent growth curves of these bacteria at 10^3 to 10^8 CFU/mL. Data are represented as mean $\pm$ SEM of two independent experiments performed in triplicate. P-values were determined by one-way ANOVA with Dunnett's multiple comparisons test relative to FSA:GM control. IL-33 measured by ELISA. FSA: *S. aureus* secretome from 6 h culture in keratinocyte growth media 2, C: keratinocyte growth media 2. Source data are provided as a Source Data File.

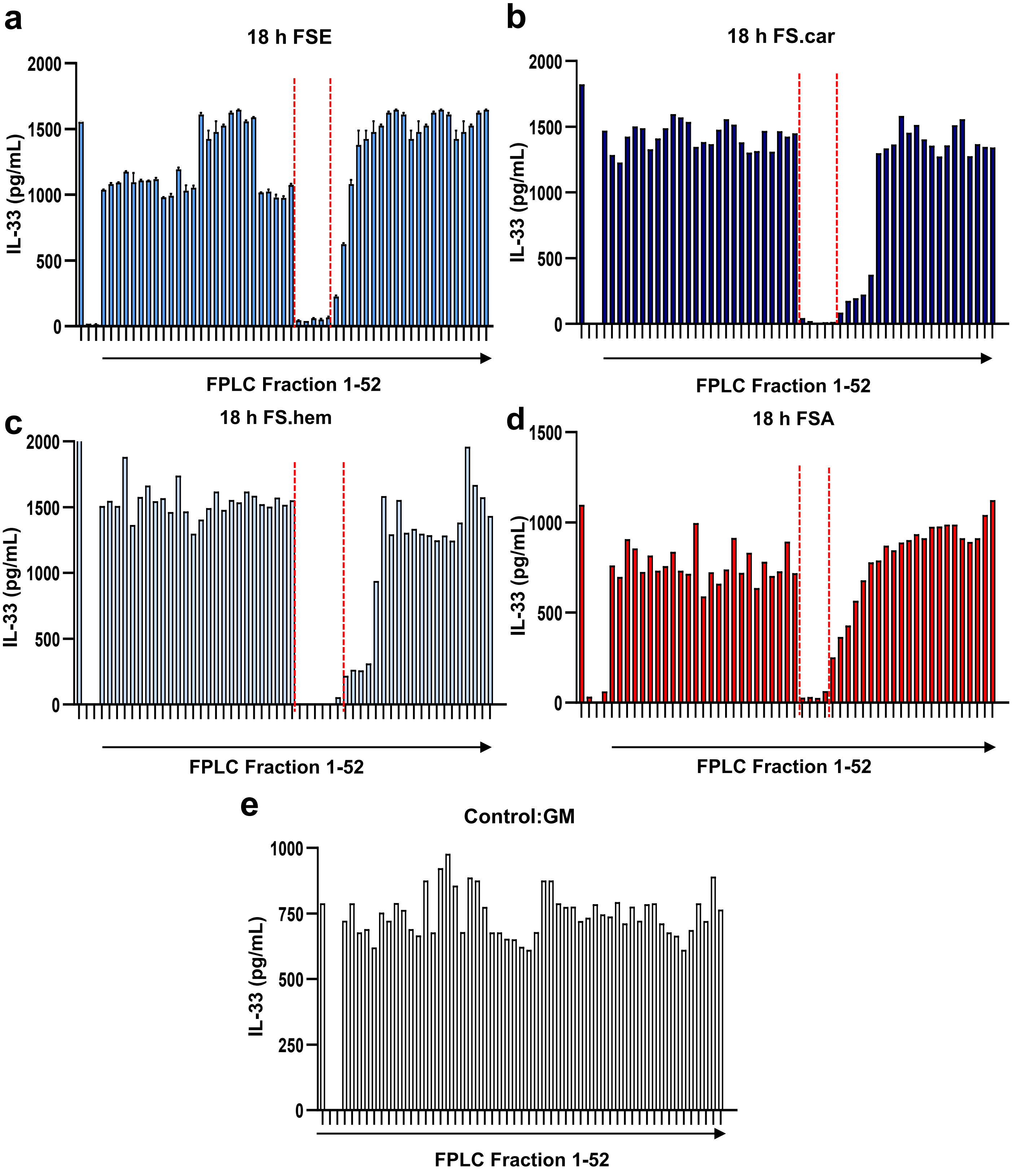
a
18 h FSE
IL-33 (pg/mL)
2000
1500
1000
500
0
FPLC Fraction 1-52
b
18 h FS.car
IL-33 (pg/mL)
2000
1500
1000
500
0
FPLC Fraction 1-52
c
18 h FS.hem
IL-33 (pg/mL)
2000
1500
1000
500
0
FPLC Fraction 1-52
d
18 h FSA
IL-33 (pg/mL)
1500
1000
500
0
FPLC Fraction 1-52
e
Control:GM
IL-33 (pg/mL)
1000
750
500
250
0
FPLC Fraction 1-52

Figure S3: Immunomodulatory factors released by stationary phase bacteria are small molecules eluting in the same FPLC fractions.

Elution of IL-33 inhibitory activity using a Superose® 12 FPLC column released by supernatant of different staphylococcal species cultured for 18 h (10^7 CFU/mL): (a) FSE: *S. epidermidis* supernatant, (b) FS.car: *S. carnosus* supernatant, (c) FS.hem: *S. haemolyticus* supernatant, (d) FSA: *S. aureus* supernatant and (e) Control: GM: Keratinocyte growth media 2. Data represents three pooled technical replicates. FPLC: Fast Liquid Protein Chromatography. IL-33 was measured by ELISA. Source data are provided as a Source Data File.

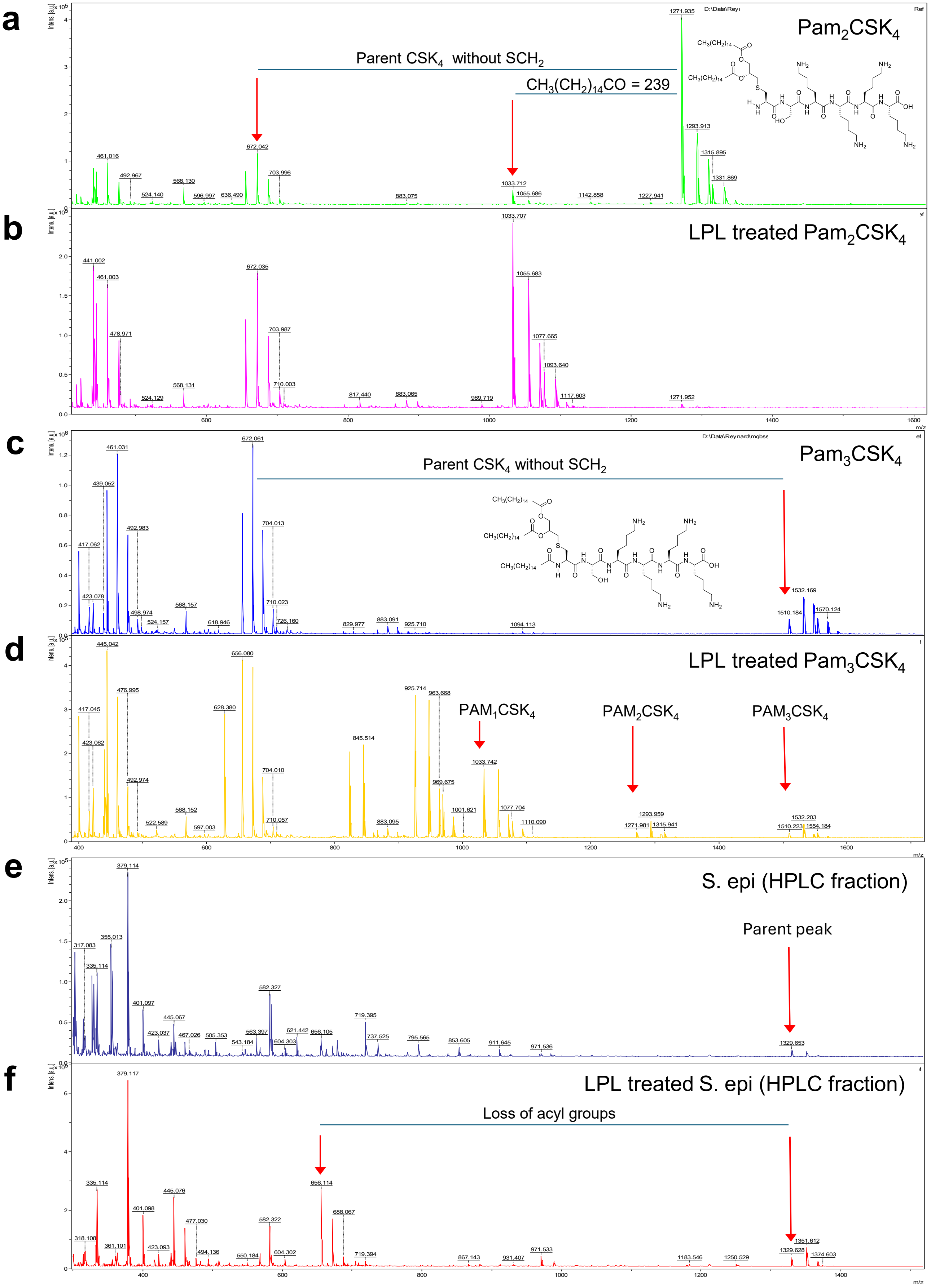
a
Pam$_2$CSK$_4$
Parent CSK$_4$ without SCH$_2$
CH$_3$(CH$_2$)$_{14}$CO = 239
b
LPL treated Pam$_2$CSK$_4$
c
Pam$_3$CSK$_4$
Parent CSK$_4$ without SCH$_2$
d
LPL treated Pam$_3$CSK$_4$
PAM$_1$CSK$_4$
PAM$_2$CSK$_4$
PAM$_3$CSK$_4$
e
S. epi (HPLC fraction)
Parent peak
f
LPL treated S. epi (HPLC fraction)
Loss of acyl groups

Figure S4: Lipoprotein lipase (LPL) treatment of bacterial lipoproteins results in loss of acyl groups by MALDI-TOF MS/MS.

Synthetic lipoproteins Pam_2CSK_4 (a) and Pam_3CSK_4 (c) or *S. epidermidis* bioactive HPLC fractions (e) were treated with lipoprotein lipase (LPL) for 30 min at 37 °C before analysis by MALDI-TOF MS/MS. LPL treatment resulted in loss of acyl groups as seen by change in MS/MS peaks (b, d, f). Red arrows denote assignment of key peaks. Data represents n=1 from three pooled technical replicates/sample. Pam_2CSK_4: synthetic diacylated lipopeptide, Pam_3CSK_4: synthetic triacylated lipopeptide. Source data are provided as a Source Data File.

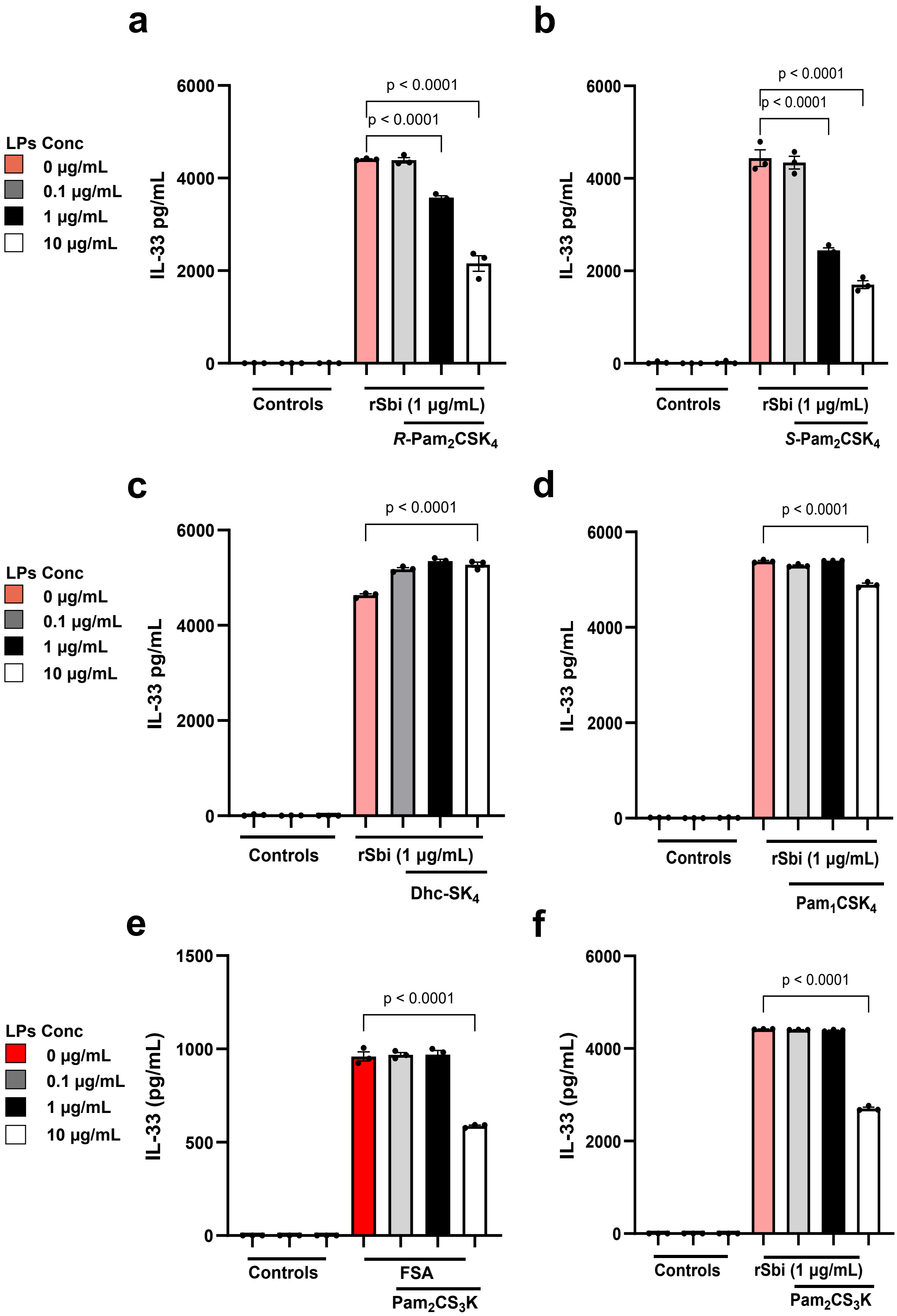

a
b
c
d
e
f
LPs Conc
0 µg/mL
0.1 µg/mL
1 µg/mL
10 µg/mL
IL-33 pg/mL
IL-33 (pg/mL)
p < 0.0001
Controls
rSbi (1 µg/mL)
R-Pam2CSK4
S-Pam2CSK4
Dhc-SK4
Pam1CSK4
FSA
Pam2CS3K

Figure S5: Immunomodulatory activity of synthetic lipopeptides differing by acyl chain number and isomer type.

Inhibitory effect on rSbi or FSA-induced IL-33 released by NHEK with addition of (a) *R*-isomer Pam_2CSK_4 (0.1-10 μg/mL); (b) *S*-isomer Pam_2CSK_4 (0.1-10 μg/mL); (c) Dhc-SK_4 (0.1-10 μg/mL); (d) Pam_1-CSK_4 (0.1-10 μg/mL); (e) Pam_2CS_3K (0.1-10 μg/mL); (f) Pam_3CS_3K (0.1-10 μg/mL). Data are represented as mean ± SEM of three independent experiments performed in triplicate. P-values were determined by one-way ANOVA with Dunnett's multiple comparisons test relative to rSbi (a, b, c, d, f) or FSA (e) only control. IL-33 was measured by ELISA. Controls represent IL-33 release from NHEK treated with GM, the synthetic lipopeptide reconstitution vehicle, or synthetic lipopeptide only. GM: keratinocyte growth media 2, Dhc: dehydrocorydaline, Pam_1CSK_4: synthetic monoacylated lipopeptide, *R*- and *S*-Pam_2CSK_4: synthetic diacylated lipopeptide, Pam_2CS_3K: synthetic diacylated lipopeptide, Pam_3CS_3K: synthetic triacylated lipopeptide. Source data are provided as a Source Data File.

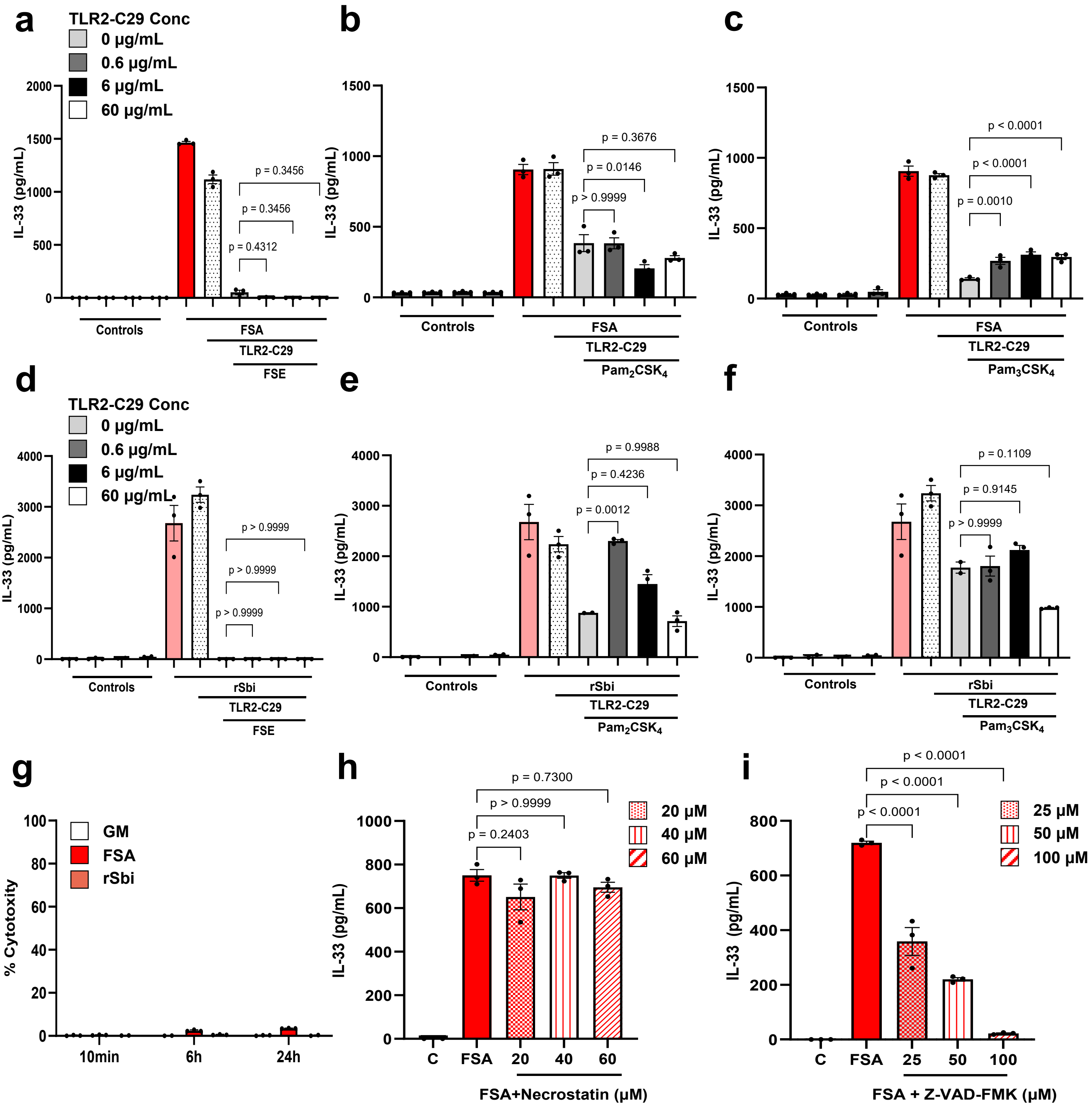

a
TLR2-C29 Conc
0 μg/mL
0.6 μg/mL
6 μg/mL
60 μg/mL
IL-33 (pg/mL)
p = 0.3456
p = 0.3456
p = 0.4312
Controls
FSA
TLR2-C29
FSE
b
IL-33 (pg/mL)
p = 0.3676
p = 0.0146
p > 0.9999
Controls
FSA
TLR2-C29
Pam2CSK4
c
IL-33 (pg/mL)
p < 0.0001
p < 0.0001
p = 0.0010
Controls
FSA
TLR2-C29
Pam3CSK4
d
TLR2-C29 Conc
0 μg/mL
0.6 μg/mL
6 μg/mL
60 μg/mL
IL-33 (pg/mL)
p > 0.9999
p > 0.9999
p > 0.9999
Controls
rSbi
TLR2-C29
FSE
e
IL-33 (pg/mL)
p = 0.9988
p = 0.4236
p = 0.0012
Controls
rSbi
TLR2-C29
Pam2CSK4
f
IL-33 (pg/mL)
p = 0.1109
p = 0.9145
p > 0.9999
Controls
rSbi
TLR2-C29
Pam3CSK4
g
% Cytotoxity
GM
FSA
rSbi
10min
6h
24h
h
IL-33 (pg/mL)
p = 0.7300
p > 0.9999
p = 0.2403
20 μM
40 μM
60 μM
C
FSA
20
40
60
FSA+Necrostatin (μM)
i
IL-33 (pg/mL)
p < 0.0001
p < 0.0001
p < 0.0001
25 μM
50 μM
100 μM
C
FSA
25
50
100
FSA + Z-VAD-FMK (μM)

Figure S6: Immunomodulatory activity of soluble lipopeptides is not abrogated by TLR2 inhibition, but by caspase inhibition.

Pre-incubation with TLR2-C29 (0–60 µg/mL) did not abrogate the inhibitory effect of FSE, Pam_2CSK_4 (10 µg/mL), or Pam_3CSK_4 (10 µg/mL) on FSA (a-c) or rSbi (d-f)-induced release of IL-33 by NHEK. Furthermore, IL-33 release from NHEK is not linked to cell death as there was no observable LDH activity in response to FSA or rSbi stimulation (g), and the RIP-kinase inhibitor necrostatin-1 (h) had no effect. However, the pan-caspase inhibitor Z-YVAD-FMK suppressed FSA-induced IL-33 release (i). Data are represented as mean ± SEM of three independent experiments performed in triplicate. P-values were determined by one-way ANOVA with Tukey's multiple comparisons test (a-f, h, i), two-way ANOVA with Tukey's multiple comparisons test (g). IL-33 was measured by ELISA. FSA 6 h *S. aureus* supernatant; FSE: 18 h *S. epidermidis* supernatant; GM: keratinocyte growth media 2; Pam_2CSK_4: synthetic diacylated lipopeptide, Pam_3CSK_4: synthetic triacylated lipopeptide. Controls represent IL-33 release from NHEK treated with either GM, the TLR ligand reconstitution vehicle, the TLR antagonist, and the TLR ligand only. Source data are provided as a Source Data File.

Table S1: List of abbreviations used in this study.

Abbreviation	Definition
AD	Atopic dermatitis
AMPs	Anti-microbial peptides
CFUs	Colony-forming units
DAPI	4’,6-diamidino-2-phenylindole
FITC	Fluorescein Isothiocyanate
FPLC	Fast liquid protein chromatography
FSA	*Staphylococcus aureus* secretome
FS.coh	*Staphylococcus cohnii* secretome
FS.cap	*Staphylococcus capitis secretome*
FS.car	*Staphylococcus carnosus* secretome
FSE	*Staphylococcus epidermidis* secretome
FS.hem	*Staphylococcus haemolyticus* secretome
FS.len	*Staphylococcus lentus secretome*
GM	Keratinocyte growth medium 2
GSDMD	Gasdermin D
HT	Heat treatment (95ᵒC)
HT LPL	Heat treated (95ᵒC) lipoprotein lipase
ICC	Immunocytochemistry
IL-33	Interleukin-33
LPL	Lipoprotein lipase
MAMPs	Microbe-associated molecular patterns
NHEK	Normal human epidermal keratinocytes
RIP1	Receptor-interacting protein 1
rSbi	Recombinant second immunoglobulin-binding protein
Sbi	Second immunoglobulin-binding protein
S. aureus	Live *Staphylococcus aureus*
S. coh	Live *Staphylococcus cohnii*
S. cap	Live *Staphylococcus capitis*
S. car	Live *Staphylococcus carnosus*
S. epi	Live *Staphylococcus epidermidis*
S. hem	Live *Staphylococcus haemolyticus*
S. len	Live *Staphylococcus lentus*
TEWL	Transepithelial water loss
TLR	Toll like receptor
TIR	Toll/interleukin-1 receptor
TSLP	Thymic stromal lymphopoietin
TY	Trypsin

Table S2: Bacterial strains and cell lines used in this study.

REAGENT	SOURCE	IDENTIFIER
**Bacterial Strains**		
S. aureus	A. McBain, University of Manchester, UK	MBRG 16.1
S. aureus	J. Geoghegan, University of Birmingham, UK	Newman (Nm)
S. epidermidis	G. Xia, University of Manchester, UK	1457
S. capitis	G. Xia, University of Manchester, UK	ATCC 27840
S. carnosus	G. Xia, University of Manchester, UK	TM300
S. cohnii	G. Xia, University of Manchester, UK	ATCC 29974
S. hemolyticus	G. Xia, University of Manchester, UK	JCSC 1435
S. lentus	G. Xia, University of Manchester, UK	3472
**Human keratinocyte cell culture**		
Normal Human Epidermal Keratinocytes (NHEK)	PromoCell	C-12002
NHEK Donors (12)	PromoCell	438Z014; 3070302.2; 3070909.2; 3070301.2; 438Z002; 474Z020.1; 488Z008.2; 494Z030.1; 488Z006.1; 494Z030.2; 503Z004.1; 454Z029
Keratinocyte Growth Medium 2 (GM) + Supplements	PromoCell	C-20011
HEPES, Buffered Saline Solution	PromoCell	C-4000
DetachKit2 Trypsin, Trypsin neutralizing solution	PromoCell	C-41212

Table S3: Information of antibodies used in this study.

Antigen	Source	Supplier	Dilution	Catalogue no.
**Antibodies for Immunocytochemistry (ICC)**				
GSDMD	Rabbit	Thermo Fisher Scientific	1:100	PA5-116815
IL-33	Mouse	Enzo Life Sciences	1:200	ALX-804-840
IgG	Mouse	Vector Laboratories	1:200	BA-9200-1.5
Strepavidin-Cy3™	*Streptomyces avidinii*	Merck Life Sciences	1:100	S6402
Cytokeratin 14	Rabbit	Thermo Fisher Scientific	1:200	PA5-28002
IgG Alexa Flour™ 488	Goat	Thermo Fisher Scientific	1:1,000	A-11008
IgG Texas Red	Goat	Thermo Fisher Scientific	1:1,000	PA5-16661
IgG Alexa Flour™ 488	Goat	Thermo Fisher Scientific	1:1,000	F-2761
ProLong™ Mountant with DAPI	n/a	Thermo Fisher Scientific	n/a	P36962
**Antibodies for Immunofluorescence (IF)**				
IL-33	Rabbit	Abcam	1:2,000	Ab187060
IgG Alexa Fluor™ 488	Goat	Invitrogen	1:1,000	A32731
Cellstain® DAPI solution	n/a	Dojindo	n/a	D523
ProLong™ Gold Antifade Mountant	n/a	Invitrogen	n/a	P36934

Table S4: Information of key reagents used in this study.

Reagent	Supplier	Catalogue no.
Recombinant *S. aureus* Immunoglobulin-binding protein sbi (rSbi)	Stratech	CSB-EP642039FLFc7-CSB-CU STOM
Human TSLP DuoSet ELISA	Bio Techne	DY1398
Lipoprotein lipase *Pseudomonas* sp.	Sigma-Aldrich®	62335
(R) Pam_2CSK_4	Tocris	4637
(R) Pam_3CSK_4	Tocris	4633
Pam-CSKKKK	EMB microcollections	L20201
R-Pam_2Cys-SKKKK	EMB microcollections	L20202
S-Pam_2Cy2-SKKKK	EMB microcollections	L2048
Pam_2Cys-SKKKK(Fluorescein-Aca-Aca)	EMB microcollections	L20214
R-Pam_3Cys-SKKKK	EMB microcollections	L2011
Dhc-SKKKK	EMB microcollections	L2001
Pam_2Cys-SSSK	CPC Scientific Inc	n/a
Pam_3Cys-SSSK	CPC Scientific Inc	n/a
FSL-1 (trifluoroacetate salt)	Cambridge Bioscience	CAY24220
Disulfiram	Stratech	A4015
LDC7559	Stratech	S9622
Ac-FLTD-CMK	Bio Techne	7242
CU-CPT22	MedChemExpress	HY-108471
TLR2-C29	Invivogen	inh-c29
Necrostatin-1 (Nec-1)	MedChemExpress	HY-15760
Z-VAD-FMK	Invivogen SAS	tlfl-vad
