# Supplementary material for: Soluble bacterial lipopeptides suppress gasdermin D-associated IL-33 release in keratinocytes and atopic dermatitis in mice
Source: Nat Commun. 2026 May 13;17:6367. doi: 10.1038/s41467-026-72376-x (PMC13377172; doi:10.1038/s41467-026-72376-x)
Supplement: Supplementary file 3 — Transparent Peer Review file [file 41467_2026_72376_MOESM3_ESM.pdf]

nature portfolio

Peer Review File

Soluble bacterial lipopeptides suppress gasdermin D-associated IL-33 release in keratinocytes and atopic dermatitis in mice

Corresponding Author: Dr Peter Arkwright

This file contains all reviewer reports in order by version, followed by all author rebuttals in order by version.

Version 0:

Reviewer comments:

Reviewer #1

(Remarks to the Author)
In this manuscript, the authors Williams et al. present a compelling investigation into the immunomodulatory role of soluble bacterial lipopeptides in the context of atopic dermatitis (AD). The study challenges the prevailing understanding of these molecules as primarily immunostimulatory by demonstrating their ability to suppress gasdermin D (GSDMD)-associated IL-33 release in keratinocytes and alleviate AD-like symptoms in a mouse model.
The study reveals that various Gram-positive and Gram-negative bacteria release soluble lipopeptides during their stationary growth phase, modulating allergic responses rather than stimulating them. This immunomodulatory activity was shown to be destroyed by lipoprotein lipase (LPL), indicating the importance of intact acyl groups. These bacterial factors, particularly diacylated lipopeptides, inhibit IL-33 and TSLP secretion by human keratinocytes in vitro and ex vivo. The study demonstrates mechanistically that these lipopeptides inhibit the active release of IL-33 from the nucleus of live keratinocytes, leading to its accumulation at the perinuclear rim. This suppression is independent of classical CD14 and TLR signaling pathways. The researchers found that Sbi from S. aureus induces IL-33 release by promoting the loss of constitutive GSDMD expression. Notably, the immunomodulatory lipopeptides (FSE, Pam2CSK4, Pam3CSK4) prevent this loss, leading to the retention of both GSDMD and IL-33 inside the cell. Furthermore, GSDMD inhibitors suppress FSA and rSbi-induced IL-33 release, indicating that lipopeptides likely exert their effect by suppressing Sbi-induced GSDMD activity.
Strengths of the Study:
• Novelty: The findings challenge the established paradigm regarding the role of bacterial lipopeptides in immune responses, presenting them as potential immunomodulatory agents rather than solely pro-inflammatory triggers.
• Comprehensive Approach: The study employs a multi-faceted approach, utilizing in vitro human keratinocyte cultures, ex vivo human skin explants, and an in vivo mouse model to support its conclusions.
• Mechanism Elucidation: The research delves into the underlying mechanisms of action, demonstrating a TLR2-independent pathway involving the suppression of caspase/GSDMD-mediated IL-33 release.
• Therapeutic Potential: The study highlights the potential of soluble bacterial lipopeptides, particularly diacylated forms, as novel therapeutic agents for allergic diseases like AD. This is further supported by the in vivo efficacy of Pam2CSK4 in the mouse model.
• Relevance to Environmental Exposure: The findings offer a molecular explanation for the observed protective effects of farm environments and exposure to bacterial products against allergic diseases.
Major/ Minor Concerns:
• Specificity of Lipopeptides: While the study shows that various bacterial species release immunomodulatory factors with similar molecular weights and LPL sensitivity, further investigation into the specific structural characteristics of these lipopeptides across different species could be beneficial. Identifying the precise molecules responsible for the observed effects would be valuable.
• In vivo Discrepancy: The finding that topical Pam2CSK4, but not Pam3CSK4, suppressed eczema in mice, despite both showing in vitro IL-33 inhibition, warrants further exploration. Factors such as skin penetration, stability, or specific receptor interactions in vivo might explain this difference. Authors should comment on the in vivo discrepancy in the discussion.
• Long-term Effects and Safety: The in vivo study lasted four weeks. Long-term studies are needed to assess these lipopeptides' sustained efficacy and safety profile as potential therapeutics.
• Mechanism Downstream of GSDMD: While the study convincingly demonstrates the role of GSDMD inhibition, further investigation into the precise molecular mechanisms by which lipopeptides suppress GSDMD activity in keratinocytes would

enhance understanding of this novel pathway.
Significance and Implications for Publication:
This study offers important new insights into the complex interplay between bacteria and the host immune system in the context of allergic skin disease. Identifying soluble bacterial lipopeptides as immunomodulatory molecules that can suppress IL-33 release through a non-classical, GSDMD-dependent pathway represents a significant contribution to the field. The findings could reshape our understanding of AD pathogenesis and pave the way for developing novel, chemically stable therapeutic strategies based on bacterial lipopeptides. The comprehensive experimental design and clear results strongly support the publication of this work.
Conclusion:
The manuscript presents a well-designed and executed study with compelling findings that challenge existing dogma and offer promising avenues for treating atopic dermatitis. The novelty of the findings, the robust experimental evidence, and the detailed mechanistic insights make this manuscript highly suitable for publication in a relevant high-impact journal. Addressing the potential weaknesses and areas for further investigation in future studies will further strengthen the impact of this research.

Reviewer #2

(Remarks to the Author)
Overall, the group led by Dr Williams provide a very interesting pre-clinical model of the treatment potential of bacterial lipopeptides for atopic dermatitis. There are a few conceptual disagreements I would make, and some clarifications needed but I think the work is overall valuable and would be well received by the AD research community.
The two main overall issues are:
1) The discussion makes too much of the connection between rising rates of AD and Staph. The issues connecting this claim are (a) Staph is contagious and AD is not, (b) anti-Staph treatments fail to treat AD, (c) anti-inflammatory treatments (dupilumab, steroids, etc) help AD and subsequently decrease Staph aureus despite no direct targeting, and (d) the farm exposures the authors mention as protective actually increase exposure to SA. So, I think they are stretching a bit by framing the opening around SA as causal. This isn't needed for their point that commensals can offer something beneficial so removing it from the intro and discussion does not invalidate their findings.
2) The paper mentions Gram negative bacteria but tests only 1 isolate of E coli that is a gut commensal and not skin. Skin Gram negatives that have been used in mouse models of AD include Nitrosomonas and Roseomonas, so using only a gut E coli is not a reasonable means of including Gram negatives in the discussion of the skin. Furthermore, "LPS" is a category of molecules, not a molecule. So using only 1 version (from a pathogen O111:B4) does not support the argument in the paper – for example here is paper outlining how different LPS's can be: https://pubs.acs.org/doi/10.1021/acs.analchem.4c01566
As such, I think the authors should remove Gram neg and LPS from the paper. They still have plenty of data on the effects of Staph species, so removing LPS/E coli does not in any way invalidate the remaining data. If the authors test actual commensal bacteria later, then they could make that a subsequent paper indicating that Gram neg also make helpful lipopeptides.
Figure 2 is the heart of the paper and very hard to argue against. I think that alone suggests that they have something promising. However, I would have liked to see skin PCR on the mice treated. If the argument is that the effect is mediated through a drop in IL-33 and alteration in GSDMD – the mRNA from the mouse skin (or staining) should support this claim. If the mouse skin does not appear to differ in IL-33 or TSLP, then it would be harder to connect the cell results to the mice (even though the mouse improvement would be no less impressive).
The experiments in Figures 1 and 3 need to have concurrent cell viability data. The LPS does they use in 3E for example is massive (we typically use 10ng). So it could be that they are killing the cells and thus seeing a decrease in IL-33 production. Similarly, Fig 1E needs a dose response for each of the soluble factors presented. Diluting these into GM should identify dose dependent inhibition. It would also indicate how much of the loss of FSA induction of IL33/TSLP is from an inhibitory signal versus the impacts of depleted media alone. Take an over-concentrated GM and diluting it back to 1x in FSE/FS len, FS hem, etc: seeing no loss in inhibition would suggest the presence of an inhibitor. If you make hyper concentrated GM and dilute it into FSE/etc, and the effects are lost, then it suggests something was metabolized out of the media rather than something being added to it by the bacteria.
The experiments in fig 6 seem like they should be repeated using FSE and the other secretions. Fig 6 shows the effects of FSA are partly blocked by GSDMD blockade. To connect back to FSE and the other commensals being invoked in the paper, they should test the effects on the secretions. Overall, the experiments showing a dose-response for the secretomes presented in 1E (minus E coli) could identify the target dose. Then, they could treat with LPL and one of the GSDMD inhibitors (I don't think they need all 3 for this point since each was the same) to show the effects are also lost. Otherwise, I think they have a case that the commensals make an LPL-sensitive substance that prevents IL-33 release but the details are entirely dependent on a synthetic LP.
Clarifications:
The paper would GREATLY benefit from a supplemental table, or included table if space allows, explaining the abbreviations in the treatments up front. For example, just looking at figure 1B, I'm reading that as saying they are stimulating with live Staph epi (SE) with either growth media (GM) or media that has had SA grown in it for 48 hours (FSA)? But in 1C, there is an FSA in media and FSA plus lice SE. I'm pretty sure I have it, but it took some work to piece together. The abbreviations don't make that obvious so maybe a figure legend early on outlining what each is. Also, FSA isn't defined until line 114 despite being in the figure (yes, it is in the legend but it's work to put together). And 1E uses labels like S epi when the legend says it is the soluble products (so I would think = FSE) where other figures use S epi to mean live. Please just clarify so that there is consistency. I get word limits are always an issue – but if the authors remove the extrapolations to the role of SA from the paper, they could have room to add 2-3 sentences at the end of the introduction providing a more general outline of their experimental approach – or add a Sup fig that clarifies.

Lines 186-195 I think are an over extrapolation of the studies in the field. The work connecting exposure to LPS on farms with AD did not indicate directionality. For example, patients with AD have reduced Gram negative bacteria (and all other commensals) due to an overgrowth of SA. That would mean that a patient with AD *should* shed less Gram negatives, LPS, and LP's than a heathy control. Thus, it could mean that patients with AD are putting less LPS into their house dust, rather than a lack of house dust LPS is giving inhabitants AD. The citation provided for farm dust inhibition of IL-33 did not have a comparison dust (ie did not show that city-dust would fail to perform the same outcomes). Similarly, the line about S aureus "mellows with age" is a bit much. Comparing Staph grown in static media for 24 hours to how Staph would behave on something far more dynamic like the skin is not a fair comparison and, importantly, is not needed to make the case the authors are making.

Overall I would judge this as a "major revision" due to the experiments needed, but I think with those experiments added it would be a very well received paper and would be valuable/significant to the community.

Reviewer #3

(Remarks to the Author)
The authors have previously shown that second immunoglobulin-binding protein (Sbi) secreted by S. aureus promotes atopic dermatitis (AD)-associated inflammation by inducing IL-33 release from keratinocytes. Now the authors report that this Sbi-induced IL-33 secretion can be inhibited by factors secreted by skin commensals (and S. aureus) when reaching their stationary growth phase. MALD-TOF MS/MS analyses indicated that the responsible factors are low molecular weight acylated lipopeptides. Accordingly, synthetic lipopeptides, especially Pam2CSK4 elicited inhibitory activity. Mechanistically, the lipopeptides inhibited Sbi-induced gasdermin D-mediated IL-33 release. In summary, this is an interesting study highlighting a novel anti-inflammatory activity of bacterial products in the context of AD.

I have only a few comments:

1. Fig. 1A. I am wondering why bacteria growth did not exceed OD 0.2. Normally, the log growth phase is in the range between OD 0.1 – 0.8 and growth exceeds an OD of 1.0 in the stationary phase after 24 h incubation time.

2. The bacterial supernatants have been produced by shaking conditions (300rpm). This is an artificial situation, which does not reflect the in vivo situation. It is known that bacteria drastically change their expression pattern dependent on the growth conditions (shaking vs. static growth conditions). Do the authors have any idea if the bacteria produce similar activity when incubated under static conditions?

3. Abstract: "modulate rather than 30 stimulate allergic responses in vitro". I suggest specifying "modulate" in this context and change it to "suppress" or "restrict" or similar words.

4. Figure 3B-E: Please explain different color of the bars.

5. Please explain the ex vivo method in more detail (which adhesive film, how many times tape stripping, how many S. epidermidis were applied)

6. Line 137: The authors state "there was no evidence of cell death…" However, in Fig. S3H significant increase of cytotoxicity was reported.

Version 1:

Reviewer comments:

Reviewer #1

(Remarks to the Author)
Williams et al. demonstrate that soluble bacterial lipopeptides from skin commensals suppress gasdermin D (GSDMD)-mediated IL-33 release from keratinocytes, thereby modulating inflammation in atopic dermatitis. Key findings include: (1) bacterial secretomes from stationary-phase cultures of various Staphylococcus species inhibit Sbi-induced IL-33 release; (2) the active factors are heat-stable, low molecular weight (<3 kDa) lipopeptides sensitive to lipoprotein lipase; (3) synthetic diacyl lipopeptides, particularly Pam2CSK4, replicate this inhibitory activity; and (4) these lipopeptides improve AD-like inflammation in mouse models through GSDMD-dependent mechanisms.

Strengths:
- The experimental design is thorough, utilizing both in vitro keratinocyte assays and in vivo AD mouse models.
- Proper controls are included consistently, such as heat-inactivated enzymes, multiple bacterial species, and cytotoxicity tests.
- The statistical analysis is rigorous, employing appropriate tests for normality, adjustments for multiple comparisons, and clear reporting of sample sizes.
- Using three different GSDMD inhibitors (disulfiram, necrosulfonamide, LDC7559) enhances the mechanistic conclusions.
- The authors have addressed reviewer concerns by adding dose-response curves and combining GSDMD inhibitors with

bacterial secretomes.

Weaknesses:
- The bacterial growth curves show unusually low OD values (~0.2) during the stationary phase, whereas OD typically exceeds 1.0 after 24 hours of incubation.
- While synthetic lipopeptides support the findings, the exact molecular identity of the native bacterial lipopeptides remains unclear.
- The mechanism connecting lipopeptide binding to GSDMD inhibition needs further clarification.

Significance

This work marks a significant advance in understanding host-microbiome interactions in atopic dermatitis. Identifying commensal-derived lipopeptides as anti-inflammatory mediators challenges the common view of bacterial products as mainly pro-inflammatory. The findings have important implications for developing microbiome-based treatments for AD and potentially other inflammatory skin conditions. Showing that both commensals and S. aureus can produce these protective factors adds complexity to our understanding of the skin microbiome's role in health and disease.

Data and Methodology

Strengths:
- Methods are clearly outlined with enough detail for others to reproduce the results.
- Mass spectrometry analysis correctly determines the molecular weight range of the active factors.
- Both chemical inhibitors and genetic approaches (GSDMD-/- mice) are used to validate key findings.
- The data availability statement demonstrates transparency.

Areas for improvement:
- More detailed characterization of native lipopeptide structures using advanced MS/MS or NMR would improve molecular identification.
- The ex vivo skin model methods could benefit from additional standardization details.
- Bacterial culture conditions that produce such low OD values should be clarified or re-examined.

Clarity and Context:

The manuscript is well-written and logically organized. The introduction offers suitable background on AD development and the role of the skin microbiome. The results are presented clearly, starting from initial observations to mechanistic studies. The discussion effectively puts the findings into context within the broader literature and notes limitations. The standardized abbreviations table (Supplementary Table I) improves clarity.

References:

The cited literature is thorough and up-to-date. The authors properly reference key studies on AD development, skin microbiome, and GSDMD biology. The discussion of how their findings connect to previous research on S. aureus in AD is well-balanced and recognizes different viewpoints in the field.

Suggested Improvements:

1. Structural characterization: Pursue more definitive structural identification of the native bacterial lipopeptides using advanced analytical techniques

2. Mechanistic detail: Investigate the receptor(s) or molecular targets through which lipopeptides inhibit GSDMD activation (e.g., TLR2 involvement)

3. Dose-response relationships: While dose-response data has been added, expand this to include EC50 values for all active compounds

4. Clinical relevance: Discuss potential translation to human studies, including whether these lipopeptides are detectable on human AD skin

5. Figure improvements:
- Clarify the color coding in all figures (the authors have addressed this for Figure 3)
- Include representative flow cytometry plots or gating strategies in supplementary data

6. Technical clarifications:
- Explain the low bacterial OD values observed
- Provide more details on tape-stripping methodology consistency

My Expertise

I have expertise in immunology, host-microbe interactions, and inflammatory skin diseases. While the mass spectrometry-

based structural characterization of lipopeptides is somewhat outside my primary expertise, I can assess the overall approach and conclusions. All other aspects of the manuscript are well within my area of competence.

Overall Assessment

This high-quality manuscript significantly advances our understanding of how commensal bacteria regulate skin inflammation. The work is technically solid, the conclusions are well-supported, and the findings have important implications for AD pathogenesis and treatment. The authors have responded to reviewer feedback and strengthened the manuscript accordingly.
With only minor revisions to address remaining technical issues and improve the structural analysis of the lipopeptides, this work will be ready for publication in Nature Communications.

Reviewer #2

(Remarks to the Author)
Most of the previous issues have been addressed. However, I still see concerns with claiming that the treatment works through IL-33. The authors presented data that their *model* is IL-33 dependent, and that FSE reduces IL-33 release in cells. But that does not mean that the treatment is IL-33 dependent. I suppose they could treat the mice with FSE and replace recombinant IL-33 to show that the effects are reduced, but even that is not the same. Without data that the IL-33 levels are reduced in the skin of the mice I don't see how the claims are supported - unless they are arguing that IL-33 is still produced but not signaling. A minimum, the authors should add a paragraph to the discussion outlining that their data is: FSE works through IL33 in cells, the mouse model is IL33 dependent, but the data for direct action on IL33 in the skin during treatment is lacking.

Reviewer #3

(Remarks to the Author)
I thank the authors for adequately addressing the open points and for submitting this improved revision.

Version 2:

Reviewer comments:

Reviewer #1

(Remarks to the Author)
The authors have submitted a substantially revised manuscript that addresses the majority of concerns raised in previous review rounds. This study presents novel and important findings demonstrating that bacterial lipopeptides from skin commensals suppress GSDMD-mediated IL-33 release from keratinocytes, challenging the conventional view that bacterial products are primarily pro-inflammatory. The work has significant implications for understanding host-microbiome interactions in atopic dermatitis and potential therapeutic development.

Major Strengths

1. Thorough Experimental Design: The combination of in vitro keratinocyte assays, ex vivo human skin explants, and in vivo NC/Tnd mouse models provides robust, multi-level evidence supporting the key findings.

2. Appropriate Controls: The inclusion of heat-inactivated enzymes, multiple bacterial species, cytotoxicity tests, and the use of three different GSDMD inhibitors (disulfiram, necrosulfonamide, LDC7559) strengthens the mechanistic conclusions.

3. Rigorous Statistical Analysis: The statistical methodology is appropriate, with normality testing using QQ residual plots, two-way ANOVA with Tukey's or Dunnett's post-hoc tests, and non-parametric alternatives (Mann-Whitney, Kruskal-Wallis) when normality could not be assumed.

4. Successful Resolution of Prior Concerns:
- Growth curves have been corrected using the BioTek LogPhase 600 reader
- New immunofluorescent staining data (Fig. 4C, 4D) directly addresses the IL-33 mechanism in vivo
- Tape-stripping methodology is now well-documented with confirmatory H&E staining
- Individual data points are now displayed in all figures

Reviewer #2

(Remarks to the Author)
My issues have been addressed with the new data.

Open Access This Peer Review File is licensed under a Creative Commons Attribution 4.0 International License, which permits use, sharing, adaptation, distribution and reproduction in any medium or format, as long as you give appropriate credit to the original author(s) and the source, provide a link to the Creative Commons license, and indicate if changes were made.
In cases where reviewers are anonymous, credit should be given to 'Anonymous Referee' and the source.
The images or other third party material in this Peer Review File are included in the article's Creative Commons license, unless indicated otherwise in a credit line to the material. If material is not included in the article's Creative Commons license and your intended use is not permitted by statutory regulation or exceeds the permitted use, you will need to obtain permission directly from the copyright holder.
To view a copy of this license, visit https://creativecommons.org/licenses/by/4.0/

Nature Communications manuscript NCOMMS-25-12764 *Soluble bacterial lipopeptides suppress gasdermin D-associated IL-33 release in keratinocytes and atopic dermatitis in mice*

Point-by-point response to reviewers' comments and suggestions

The authors thank the editor and reviewers very much for their encouraging and helpful feedback. Point-by-point responses to reviewers' comments are detailed below.

Editorial guidelines

Response: As requested by the Editor, we have checked and revised the manuscript formatting to meet the Journal requirements, including curtailing the word count of the Abstract and Results section subheadings. We believe we have comprehensively addressed the reviewers' comments, now including additional experiments to determine the finer structure of the immunomodulatory lipopeptides, dose response curves of all the staphylococcal commensals, and cytotoxicity experiments. We have clarified the mechanism of action of the three gasdermin D inhibitors we used, and as requested combined a GSDMD inhibitor (Disulfiram) with *S. epidermidis* secretome (FSE). We have also standardised the abbreviations throughout the manuscript. These changes are tracked within the manuscript.

Reviewer #1:

In this manuscript, the authors Williams et al. present a compelling investigation into the immunomodulatory role of soluble bacterial lipopeptides in the context of atopic dermatitis (AD). The study challenges the prevailing understanding of these molecules as primarily immunostimulatory by demonstrating their ability to suppress gasdermin D (GSDMD)-associated IL-33 release in keratinocytes and alleviate AD-like symptoms in a mouse model. The study reveals that various Gram-positive and Gram-negative bacteria release soluble lipopeptides during their stationary growth phase, modulating allergic responses rather than stimulating them. This immunomodulatory activity was shown to be destroyed by lipoprotein lipase (LPL), indicating the importance of intact acyl groups. These bacterial factors, particularly diacylated lipopeptides, inhibit IL-33 and TSLP secretion by human keratinocytes in vitro and ex vivo. The study demonstrates mechanistically that these lipopeptides inhibit the active release of IL-33 from the nucleus of live keratinocytes, leading to its accumulation at the perinuclear rim. This suppression is independent of classical CD14 and TLR signaling pathways. The researchers found that Sbi from S. aureus induces IL-33 release by promoting the loss of constitutive GSDMD expression. Notably, the immunomodulatory lipopeptides (FSE, Pam2CSK4, Pam3CSK4) prevent this loss, leading to the retention of both GSDMD and IL-33 inside the cell. Furthermore, GSDMD inhibitors suppress FSA and rSbi-induced IL-33 release, indicating that lipopeptides likely exert their effect by suppressing Sbi-induced GSDMD activity.

Strengths of the Study:

• Novelty: The findings challenge the established paradigm regarding the role of bacterial lipopeptides in immune responses, presenting them as potential immunomodulatory agents rather than solely pro-inflammatory triggers.

• Comprehensive Approach: The study employs a multi-faceted approach, utilizing in vitro human keratinocyte cultures, ex vivo human skin explants, and an in vivo mouse model to support its conclusions.

• Mechanism Elucidation: The research delves into the underlying mechanisms of action, demonstrating a TLR2-independent pathway involving the suppression of caspase/GSDMD-mediated IL-33 release.

• Therapeutic Potential: The study highlights the potential of soluble bacterial lipopeptides, particularly diacylated forms, as novel therapeutic agents for allergic diseases like AD. This is further supported by the in vivo efficacy of Pam2CSK4 in the mouse model.

• Relevance to Environmental Exposure: The findings offer a molecular explanation for the

observed protective effects of farm environments and exposure to bacterial products against allergic diseases.
Response: We thank the reviewer for their very detailed and positive comments about our manuscript.

Major/ Minor Concerns:
• Specificity of Lipopeptides: While the study shows that various bacterial species release immunomodulatory factors with similar molecular weights and LPL sensitivity, further investigation into the specific structural characteristics of these lipopeptides across different species could be beneficial. Identifying the precise molecules responsible for the observed effects would be valuable.
Response: Thank you for this suggestion. As requested, we have conducted additional in vitro experiments using structurally well characterised synthetic lipopeptides (data now shown in Supplementary Fig. 5). As lipoprotein lipase completely abrogated immunomodulatory activity when tested with FSA as a stimulant and our *in vivo* NC mouse experiments indicated that the predominant activity was with the diacyl lipopeptides, we focused on determining the minimal number of acyl chains required for bioactivity in our *in vitro* system. We found that the diacyl lipopeptide Pam_2CSK_4 of both the R and S isoform were active, while lipopeptides with mono-acylated and alkaloid- side chains were without activity. We highlight these additional findings in the Results and the Discussion sections.

• In vivo Discrepancy: The finding that topical Pam2CSK4, but not Pam3CSK4, suppressed eczema in mice, **despite both showing in vitro IL-33 inhibition**, warrants further exploration. Factors such as skin penetration, stability, or specific receptor interactions in vivo might explain this difference. **Authors should comment on the in vivo discrepancy in the discussion**.
Response: Thank you for raising this point. As requested, we have added a comment within the Discussion section and supportive references regarding this discrepancy and the likely explanation: penetration of drugs through the stratum corneum of the epidermis is known to depend on chemical size and hydrophobicity – diacyl lipopeptides being smaller and less hydrophobia than tri-acyl lipopeptides are thus more likely to penetrate the inert barrier despite similar bioactivity in the in vitro cell line experimental system. In addition, we make the reader aware that although staphylococci can synthesise both di- and tri-acyl lipopeptides, the shift from bacterial proliferative to stationary growth phase is known to be associated with predominance of diacyl over tri-acyl structures.

• Long-term Effects and Safety: The in vivo study lasted four weeks. Long-term studies are needed to assess these lipopeptides' sustained efficacy and safety profile as potential therapeutics.
Response: The 4-week mouse study we conducted in these relatively short-lived animals parallels standard 4-month clinical trials in human subjects who have a much longer lifespan. The reviewer is correct that longer term studies are important once a specific therapeutic formulation has been determined. In our opinion and as already mentioned in the last paragraph of the Discussions section, short-term studies in humans are now indicated to confirm therapeutic efficacy before longer-term studies in the real-world can be contemplated.

• Mechanism Downstream of GSDMD: While the study convincingly demonstrates the role of GSDMD inhibition, further investigation into the **precise molecular mechanisms by which lipopeptides suppress GSDMD activity in keratinocytes** would enhance understanding of this novel pathway.
Response: Our immunocytochemistry results suggest that rather than the effects being downstream, the immunosuppressive lipopeptides block initial release of IL-33 by Sbi from the nucleus (Fig. 6). This is in keeping with our results which have already examined different points in the activation GSDMD pathway using the following inhibitors: z-VAD-FMF

(caspase inhibitor) (Suppl Fig 6I), Ac-FLTD-CMK (binds the active site of caspases 1, 5 and 4 preventing cleavage of GSDMD), LDC7559 (binds directly to GSDMD preventing activation and subsequent insertion into the cell membrane), Disulfiram (prevent the formation of GSDMD-NT pores in the cell membrane) (Fig. 6F-H). Our results are in keeping with an upstream inhibition of GSDMD activation, and thus inhibition of IL-33 release from the nucleus. These points have now been made clearer in the Results and Discussion sections.

Significance and Implications for Publication:
This study offers important new insights into the complex interplay between bacteria and the host immune system in the context of allergic skin disease. Identifying soluble bacterial lipopeptides as immunomodulatory molecules that can suppress IL-33 release through a non-classical, GSDMD-dependent pathway represents a significant contribution to the field. The findings could reshape our understanding of AD pathogenesis and pave the way for developing novel, chemically stable therapeutic strategies based on bacterial lipopeptides. The comprehensive experimental design and clear results strongly support the publication of this work.
Response: We thank the reviewer for their encouraging appraisal of our manuscript.

Conclusion:
The manuscript presents a well-designed and executed study with compelling findings that challenge existing dogma and offer promising avenues for treating atopic dermatitis. The novelty of the findings, the robust experimental evidence, and the detailed mechanistic insights make this manuscript highly suitable for publication in a relevant high-impact journal. Addressing the potential weaknesses and areas for further investigation in future studies will further strengthen the impact of this research.
Response: We thank the reviewer for their encouraging appraisal of our manuscript.

Reviewer #2 (Remarks to the Author):

Overall, the group led by Dr Williams provide a very interesting pre-clinical model of the treatment potential of bacterial lipopeptides for atopic dermatitis. There are a few conceptual disagreements I would make, and some clarifications needed but I think the work is overall valuable and would be well received by the AD research community.
Response: We thank the reviewer for their encouraging appraisal of our manuscript.

The two main overall issues are:
1) The discussion makes too much of the connection between rising rates of AD and Staph. The issues connecting this claim are (a) Staph is contagious and AD is not, (b) anti-Staph treatments fail to treat AD, (c) anti-inflammatory treatments (dupilumab, steroids, etc) help AD and subsequently decrease Staph aureus despite no direct targeting, and (d) the farm exposures the authors mention as protective actually increase exposure to SA. So, I think they are stretching a bit by framing the opening around SA as causal. This isn't needed for their point that commensals can offer something beneficial so removing it from the intro and discussion does not invalidate their findings.
Response: Your points are well taken. We have modified the first paragraph in the introduction to highlight that *Staphylococcus aureus* may cause eczema flares in a disease where the primary defect is a skin barrier defect.

2) The paper mentions Gram negative bacteria but tests only 1 isolate of E coli that is a gut commensal and not skin. Skin Gram negatives that have been used in mouse models of AD include Nitrosomonas and Roseomonas, so using only a gut E coli is not a reasonable means of including Gram negatives in the discussion of the skin. Furthermore, "LPS" is a category of molecules, not a molecule. So using only 1 version (from a pathogen O111:B4) does not support the argument in the paper – for example here is paper outlining how

different LPS's can be: https://pubs.acs.org/doi/10.1021/acs.analchem.4c01566

As such, I think the authors should remove Gram neg and LPS from the paper. They still have plenty of data on the effects of Staph species, so removing LPS/E coli does not in any way invalidate the remaining data. If the authors test actual commensal bacteria later, then they could make that a subsequent paper indicating that Gram neg also make helpful lipopeptides.
Response: As requested, we have taken the reviewer's comment on board and removed the *E. coli* and LPS data from the text and figures of our paper. As suggested, effect of Gram-negative bacteria and LPS is something we can pursue in a subsequent study.

Figure 2 is the heart of the paper and very hard to argue against. I think that alone suggests that they have something promising. However, I would have liked to see skin PCR on the mice treated. If the argument is that the effect is mediated through a drop in IL-33 and alteration in GSDMD – the mRNA from the mouse skin (or staining) should support this claim. If the mouse skin does not appear to differ in IL-33 or TSLP, then it would be harder to connect the cell results to the mice (even though the mouse improvement would be no less impressive).
Response: As we are investigating release of constitutive IL-33 from the nucleus over a 6-hour time period rather than increased *de novo* production, we would not expect an increase in IL-33 mRNA in the skin of our NC mouse model. For the reviewer's information, we have looked at IL-33 mRNA by PCR in skin biopsies from the NC mice treated for 4 weeks with SDS only (A), FSA (B), addition of FSE alone (C) or in combination with FSA (D) (see figure below). We find no differences in IL-33 mRNA measured by qPCR.

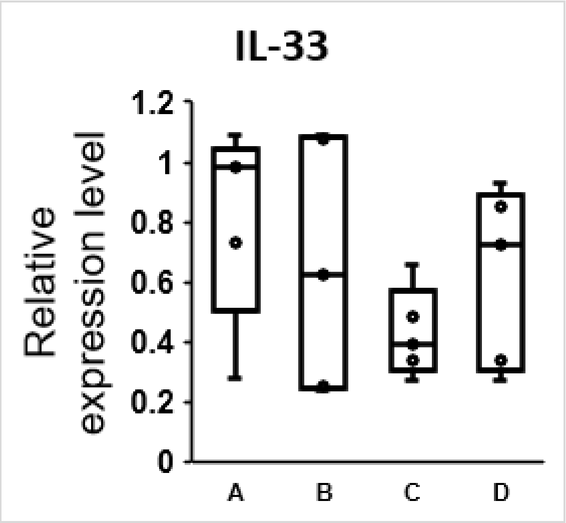

The seminal importance of IL-33 in the pathogenesis of AD has already been shown our previous publication (Fig. 7, Al Kindi *et al*, 2020 - DOI: 10.1016/j.jaci.2020.09.023) (figure detailed below). In the NC mouse model we have demonstrated that subcutaneous anti-IL-33 monoclonal antibody administration completely abrogates the eczema (both in terms of clinical score and scratching frequency) as well as TEWL in this model.

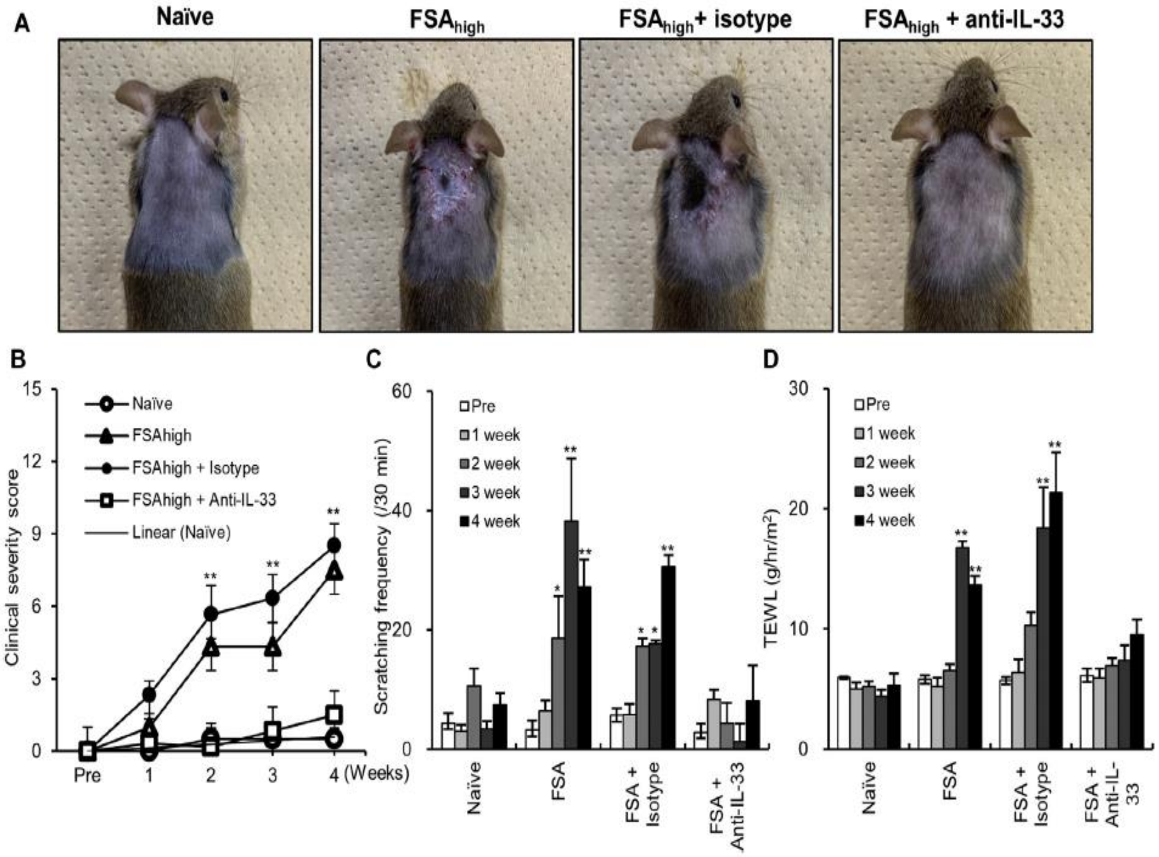

FIG 7. FSA-induced eczema in the NC/Tnd mouse can be completely abrogated by neutralizing anti–IL-33 antibodies. Eighty to hundred microliter of high (10^8 CFU/mL) concentration FSA with concomitant 4% SDS was applied topically to the backs of 8-week-old NC/Tnd mice each day for 4 weeks. One group also received an intraperitoneal 10-μg injection of anti–IL-33 mAb with the first application of FSA, while a second group received an injection of an isotype control antibody. **A**, Representative images of NC/Tnd mice after application of 4% SDS and FSA, FSA daily for 4 weeks, with or without isotype, or anti–IL-33 mAb. **(B)** Clinical eczema scores, **(C)** scratching frequency, and **(D)** TEWL of naive mice, and mice treated with 4% SDS and FSA, isotype control, or anti–IL-33 antibody. All data show results of 6 individual mice/group. Mean ± SEM. $^{**}P < .01$ compared with pretreatment or the naive group.

The experiments in Figures 1 and 3 need to have concurrent cell viability data. The LPS does they use in 3E for example is massive (we typically use 10ng). So it could be that they are killing the cells and thus seeing a decrease in IL-33 production.
Response: As requested, we have added in data from additional experiments regarding cell viability showing no significant cytotoxicity of the staphylococcal commensals, their soluble components or the synthetic lipopeptide used in this study (data added as Supplementary Fig. 1C-E, Supplementary Fig. 6E).

Similarly, Fig 1E needs a dose response for each of the soluble factors presented. Diluting these into GM should identify dose dependent inhibition. It would also indicate how much of the loss of FSA induction of IL33/TSLP is from an inhibitory signal versus the impacts of depleted media alone. Take an over-concentrated GM and diluting it back to 1x in FSE/FS len, FS hem, etc: seeing no loss in inhibition would suggest the presence of an inhibitor. If you make hyper concentrated GM and dilute it into FSE/etc, and the effects are lost, then it suggests something was metabolized out of the media rather than something being added to it by the bacteria.
Response: We have now performed a dose response for each live bacteria against FSA and measured IL-33 release with concurrent growth curves (added as Supplementary Fig. 2). All but *S. carnosus* showed potent immune modulation from 10^3 – 10^8 CFU/mL. In S,

carnosus the difference in dose response fits with its differing growth curve characteristics compared with other species (now highlighted in Fig. 1E insert).

The experiments in fig 6 seem like they should be repeated using FSE and the other secretions. Fig 6 shows the effects of FSA are partly blocked by GSDMD blockade. To connect back to FSE and the other commensals being invoked in the paper, they should test the effects on the secretions. Overall, the experiments showing a dose-response for the secretomes presented in 1E (minus E coli) could identify the target dose. Then, they could treat with LPL and one of the GSDMD inhibitors (I don't think they need all 3 for this point since each was the same) to show the effects are also lost. Otherwise, I think they have a case that the commensals make an LPL-sensitive substance that prevents IL-33 release but the details are entirely dependent on a synthetic LP.
Response: As requested, we now have repeated the experiments, adding in FSA / rSbi + the GSDMD inhibitor Disulfiram (Fig. 6H). As FSE already completely suppressed rSbi induced IL-33 release, Disulfiram had no additional suppressive effect, but it also did not block its effect.

Clarifications:
The paper would GREATLY benefit from a supplemental table, or included table if space allows, explaining the **abbreviations in the treatments** up front. For example, just looking at figure 1B, I'm reading that as saying they are stimulating with live Staph epi (SE) with either growth media (GM) or media that has had SA grown in it for 48 hours (FSA)? But in 1C, there is an FSA in media and FSA plus lice SE. I'm pretty sure I have it, but it took some work to piece together. The abbreviations don't make that obvious so maybe a figure legend early on outlining what each is. Also, FSA isn't defined until line 114 despite being in the figure (yes, it is in the legend but it's work to put together). And 1E uses labels like S epi when the legend says it is the soluble products (so I would think = FSE) where other figures use S epi to mean live. Please just clarify so that there is consistency. I get word limits are always an issue – but if the authors remove the extrapolations to the role of SA from the paper, they could have room to add 2-3 sentences at the end of the introduction providing a more general outline of their experimental approach – or add a Sup fig that clarifies.
Response: Thank you for your useful comment which will help to make our complex set of results clearer. As requested, we have standardized the abbreviations used in the text and figures of the paper, replacing SE with *S. epi* and SA with *S. aureus* in Fig. 1 for better clarity, standardised the colour coding in all the figures adding legends, as well as a supplementary Table I with abbreviations (also detailed below).

Abbreviation	Definition
AD	Atopic dermatitis
FSA	*Staphylococcus aureus* secretome
FS.coh	*Staphylococcus cohnii* secretome
FS.cap	*Staphylococcus capitis secretome*
FS.car	*Staphylococcus carnosus* secretome
FSE	*Staphylococus epidermidis* secretome
FS.hem	*Staphylococcus haemolyticus* secretome
FS.len	*Staphylococcus lentus secretome*
GM	Keratinocyte growth medium 2
GSDMD	Gasdermin D
HT	Heat treatment (95°C)
HT LPL	Heat treated (95°C) lipoprotein lipase
LPL	Lipoprotein lipase
rSbi	Recombinant second immunoglobulin-binding protein
Sbi	Second immunoglobulin-binding protein
S. aureus	Live *Staphylococcus aureus*
S. coh	Live *Staphylococcus cohnii*

S. cap	Live *Staphylococcus capitis*
S. car	Live *Staphylococcus carnosus*
S. epi	Live *Staphylococcus epidermidis*
S. hem	Live *Staphylococcus haemolyticus*
S. len	Live *Staphylococcus lentus*
TEWL	Trans-epithelial water loss
TY	Trypsin

Lines 186-195 I think are an over extrapolation of the studies in the field. The work connecting exposure to LPS on farms with AD did not indicate directionality. For example, patients with AD have reduced Gram negative bacteria (and all other commensals) due to an overgrowth of SA. That would mean that a patient with AD *should* shed less Gram negatives, LPS, and LP's than a heathy control. Thus, it could mean that patients with AD are putting less LPS into their house dust, rather than a lack of house dust LPS is giving inhabitants AD. The citation provided for farm dust inhibition of IL-33 did not have a comparison dust (ie did not show that city-dust would fail to perform the same outcomes). Similarly, the line about S aureus "mellows with age" is a bit much. Comparing Staph grown in static media for 24 hours to how Staph would behave on something far more dynamic like the skin is not a fair comparison and, importantly, is not needed to make the case the authors are making.
Response: As requested in your comment above, we have removed the *E. coli* and LPS data from the text and figures of our paper. As suggested, effect of Gram-negative bacteria and LPS is something we can pursue in a subsequent study. We have also removed the comment "mellows with age".

Overall I would judge this as a "major revision" due to the experiments needed, but I think with those experiments added it would be a very well received paper and would be valuable/ significant to the community.
Response: We thank the reviewer for their encouraging appraisal of our manuscript.

Reviewer #3:

The authors have previously shown that second immunoglobulin-binding protein (Sbi) secreted by S. aureus promotes atopic dermatitis (AD)-associated inflammation by inducing IL-33 release from keratinocytes. Now the authors report that this Sbi-induced IL-33 secretion can be inhibited by factors secreted by skin commensals (and S. aureus) when reaching their stationary growth phase. MALD-TOF MS/MS analyses indicated that the responsible factors are low molecular weight acylated lipopeptides. Accordingly, synthetic lipopeptides, especially Pam2CSK4 elicited inhibitory activity. Mechanistically, the lipopeptides inhibited Sbi-induced gasdermin D-mediated IL-33 release. In summary, this is an interesting study highlighting a novel anti-inflammatory activity of bacterial products in the context of AD.

I have only a few comments:

1. Fig. 1A. I am wondering why bacteria growth did not exceed OD 0.2. Normally, the log growth phase is in the range between OD 0.1 – 0.8 and growth exceeds an OD of 1.0 in the stationary phase after 24 h incubation time.
Response: We have repeated the growth curves of *S. aureus* and *S. epidermidis* using a newer BioTek LogPhase 600 reader and found OD values to be in line with the reviewer's comment (Figure below). As such, Fig 1A has been replaced. It should be noted that in all our studies, calibration curves to determine concentration of bacteria added were done using the Miles Misra assay, not OD, and therefore the low OD would not have affected other results**.** The growth curves are shown in Figure 1E inset and Supplementary Figure 2 to demonstrate the timing of growth phases for each of the species.

A: **BioTek PowerWave XS Microplate Reader Microbiology Reader**

B: **BioTek LogPhase 600**

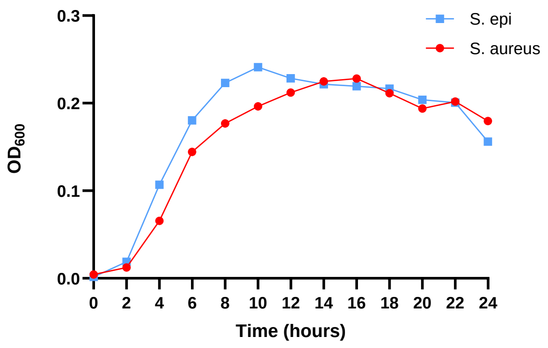

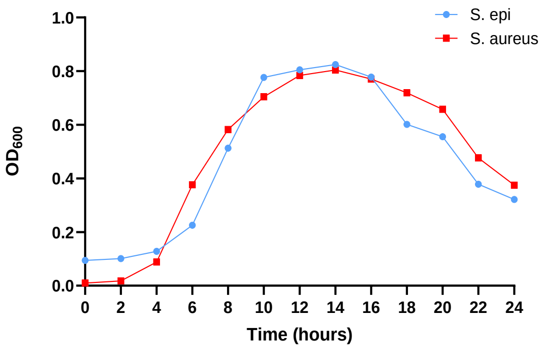

2. The bacterial supernatants have been produced by shaking conditions (300rpm). This is an artificial situation, which does not reflect the in vivo situation. It is known that bacteria drastically change their expression pattern dependent on the growth conditions (shaking vs. static growth conditions). Do the authors have any idea if the bacteria produce similar activity when incubated under static conditions?
Response: We have tested the activity of FSE when prepared using shake and static flask cultivation methods. Both methods of FSE production led to complete inhibition of FSA activity. We have just added a comment to the Results section, but we also provided a figure with the data below for the reviewer's information.

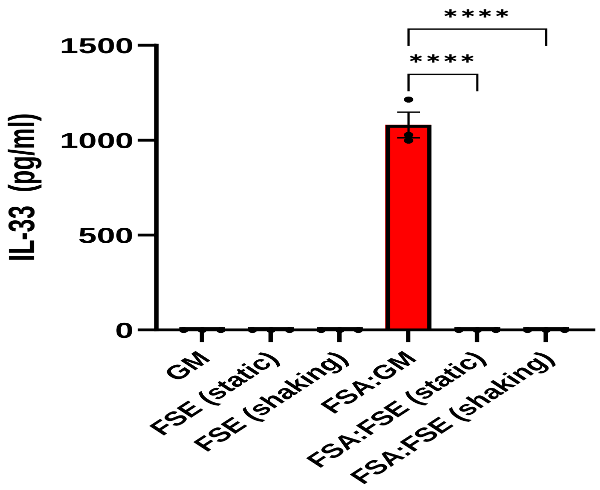

3. Abstract: "modulate rather than stimulate allergic responses in vitro". I suggest specifying "modulate" in this context and change it to "suppress" or "restrict" or similar words.
Response: The term "modulate" has been replaced by "suppress" as suggested.

4. Figure 3B-E: Please explain different color of the bars.
Response: We apologise for any confusion. We have now standardised the colours in this figure. FSA: red; FSE or other commensals: blue; FSA + FSE or other commensals: blue hatched: synthetic lipopeptides: grey.

5. Please explain the ex vivo method in more detail (which adhesive film, how many times tape stripping, how many S. epidermidis were applied).
Response: As requested, we have added more information to this part of the Methods section, namely that (1) 8 consecutive tape strips using 3.8cm^2 D-Squame® after leaving the

tape in place for 10 seconds and that 10^7CFU/ml of S. epi (live *S. epidermidis*) were used.

6. Line 137: The authors state “there was no evidence of cell death…” However, in Fig. S3H significant increase of cytotoxicity was reported.
Response: We have conducted experiments using a growth media (GM) control at each time point for comparison with the percentage of background cell death. All conditions led to 96%+ cell survival with no evidence of significant cytotoxicity by FSA or Sbi (Supplementary Fig. 6G).

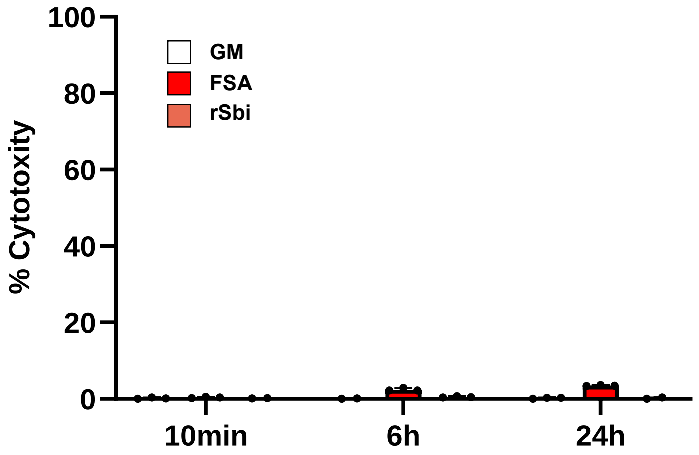

Nature Communications manuscript NCOMMS-25-12764 *Soluble bacterial lipopeptides suppress gasdermin D-associated IL-33 release in keratinocytes and atopic dermatitis in mice*

Point-by-point response to reviewers' comments and suggestions

The authors would like to thank the editor and reviewers very much for their further encouraging and thought-provoking feedback. We have conducted further extensive experiments, which we hope answer your pertinent and useful comments and suggestions. Point-by-point responses are detailed below.

Editor

As you will see from the reports below, there are outstanding concerns that we must ask you to address. In particular, Reviewer 1 reiterates that a more detailed structural characterisation of the lipopeptides is needed, as well as clarification on the bacterial growth curves and the methodology of the in vivo experimental work. Additionally, Reviewer 2 highlights that caution is needed when interpreting the IL-33-dependent mechanism on skin.

Response:

- STRUCTURAL CHARACTERISATION OF LIPOPEPTIDES
- Our MALDI-TOF and HPLC data together demonstrate that the active fractions we have isolated are likely mixtures of lipopeptides with varying efficacy. Gram positive bacteria including staphylococci synthesise a many different lipopeptides which are released as part of bacterial cell wall turnover. *S. aureus* alone synthesises 70 different lipopeptides. Combining our additional experiments and the interpretations added to the Discussion section, our data now demonstrate that there is structural diversity but functional convergence with these molecules. Specifically:
    - a minimum of 2 acyl groups are required for immunosuppressive activity *in vitro*, as non- and monoacyl lipopeptides are not bioactive.
    - Using clues from the MALDI-TOF of the peptide structure, we have now compared a Pam_2CS_3K with the standard Pam_2CSK_4 and demonstrate redundancy in the peptide sequence required for activity.
    - We find that both R- and S-diacyl structures are active, supporting a non-TLR mechanism of action, as it is already known that only the R-isomer can interact with and activate TLR2.
- These additional functional experiments complement and extend the MADI-TOF MS data. NMR analysis was not possible because of the mix of structures and their low concentrations.

- CLARIFICATION REGARDING GROWTH CURVES
- Further clarification has been added regarding the growth curves which were assessed using the Miles & Misra Method, rather than just OD.

- INTERPRETING IL-33 DEPENDENT MECHANISM
- We conducted extra experiments from skin biopsies of the NC/Tnd mice to address the comment from reviewer 2 regarding functional importance of IL-33. Results of these new experiments have been added as Fig. 4C & 4D. Immunofluorescent staining shows that IL-33 expression in the mouse epidermal skin is markedly reduced in FSA-stimulated NC/Tnd mice following treatment with both 18h FSE and the synthetic lipopeptide Pam_2CSK_4 compared to mice stimulated with FSA alone. Furthermore, in keeping with our *in vitro* data using NHEK, we also demonstrate that the reduced IL-33 expression by lipopeptides is negated by treating the 18h FSE with LPL (Fig. 4C). Together, these additional results provide further evidence for the role of IL-33 in our mouse eczema model. A comment to this effect has now also been added to the discussion (lines 174-176).

* Please replace your bar graphs with plots that feature information about the distribution of the underlying data. All data points should be shown for plots with a sample size less than 10. For larger

sample sizes, please consider box-and-whisker or violin plots as alternatives. Measures of centrality, dispersion and/or error bars should be plotted and described in the figure legend.
Response: As requested, we have now added distribution of individual data points to all figures.

Reviewer #1:
Williams et al. demonstrate that soluble bacterial lipopeptides from skin commensals suppress gasdermin D (GSDMD)-mediated IL-33 release from keratinocytes, thereby modulating inflammation in atopic dermatitis. Key findings include: (1) bacterial secretomes from stationary-phase cultures of various Staphylococcus species inhibit Sbi-induced IL-33 release; (2) the active factors are heat-stable, low molecular weight (<3 kDa) lipopeptides sensitive to lipoprotein lipase; (3) synthetic diacyl lipopeptides, particularly Pam2CSK4, replicate this inhibitory activity; and (4) these lipopeptides improve AD-like inflammation in mouse models through GSDMD-dependent mechanisms.

Strengths:
- The experimental design is thorough, utilizing both in vitro keratinocyte assays and in vivo AD mouse models.
- Proper controls are included consistently, such as heat-inactivated enzymes, multiple bacterial species, and cytotoxicity tests.
- The statistical analysis is rigorous, employing appropriate tests for normality, adjustments for multiple comparisons, and clear reporting of sample sizes.
- Using three different GSDMD inhibitors (disulfiram, necrosulfonamide, LDC7559) enhances the mechanistic conclusions.
- The authors have addressed reviewer concerns by adding dose-response curves and combining GSDMD inhibitors with bacterial secretomes.
Response: We thank the reviewer for their encouraging appraisal of our manuscript.

Weaknesses:
- The bacterial growth curves show unusually low OD values (~0.2) during the stationary phase, whereas OD typically exceeds 1.0 after 24 hours of incubation.
Response: We apologise if this was missed in our previous rebuttal, but we did repeat the growth curves of *S. aureus* and *S. epidermidis* using a newer BioTek LogPhase 600 reader and found OD values to be in line with the reviewer's comment. As such, Fig. 1A has been replaced. We have also added growth curves calculated on the BioTek reader to Supplementary Fig. S2 to demonstrate this consistency. It is important to note that in all our experiments, calibration curves to determine concentration of bacteria added were done using the Miles & Misra Method, not OD**.** This is clarified in the Methods section (line 263) and has also been added to the Fig. 1 legend.

- While synthetic lipopeptides support the findings, the exact molecular identity of the native bacterial lipopeptides remains unclear.
Response: Our data indicate that the immunosuppressive activity is not unique to one lipopeptide structure. Our functional experiments provide precise structural-functional correlates, and support the MALDI-TOF MS data that can only provide molecular weight estimates, with interpretation complicated by the fact that FSE is a mixture of structures. Taken overall, our study provides evidence for key structural elements critical for bioactivity, namely that:

1. Two or more acyl groups are an essential for activity:
    a. diacylated Pam_2CSK_4 and triacylated Pam_3CSK_4 are both immunomodulatory *in vitro*
    b. bioactivity is completely abrogated by removal of all acyl groups by LPL (Fig. 3A, 3E); confirmed by MALDI-TOF mass spectroscopy (Supplementary Fig. S4)
    c. pure lipopeptides with no (Dhc-SK_4) or one acyl chain (Pam_1CSK_4) have no activity (Supplementary Fig. S5C, S5D).

2. Activity is not restricted to one peptide structure as both diacylated Pam_2CSK_4 and Pam_2CGDPKHPKSF (FSL-1)) lipopeptides are immunomodulatory (Fig. 3B-C). Analysis of the MALDI-TOF data suggested that one of the FSE lipopeptides had a CS_3K peptide sequence. We tested this structure and found it also to have immunosuppressive properties (Fig. S5E, S5F, lines 113-115).
3. Activity is not restricted to a particularly structural isomer as additional experiments demonstrate that both R- and S-isomers of the Pam_2CSK_4 lipopeptide are immunosuppressive (Supplementary Fig. S5A, S5B). The result helps to rule out a TLR2 driven mechanism of immune regulation as previously published studies have shown that R- but not S-isomers are able to bind and activate TLR2. This structural detail is not possible using MALDI-TOF MS.
4. Skin permeation is an additional consideration for *in vivo* activity. Our *in vivo* NC/Tnd mouse model demonstrates that although both Pam_2CSK_4 and Pam_3CSK_4 reduce epidermal hyperplasia (Fig. 4A, 4B), Pam_2CSK_4 is more effective in suppressing epidermal IL-33 expression (Fig. 4C, 4D), clinical eczema (Fig. 4E) and scratching behavior (Fig. 4F). We hypothesize that a reason for this is the difference in hydrophobicity between the di- and triacylated peptides and their relative ability to penetrate the stratum corneum. Further work is required to confirm this. These findings have now also been highlighted in the Discussion section (lines 191-193).

- The mechanism connecting lipopeptide binding to GSDMD inhibition needs further clarification.
Response: In this revised manuscript, we add immunocytochemical staining of NHEK utilising FITC-labelled Pam_2CSK_4 to study its uptake and colocalization with GSDMD. We demonstrate colocalization of the FITC-Pam_2CSK_4 and GSDMD at the perinuclear rim, providing (1) evidence for uptake of lipopeptides into the keratinocytes / no evidence of binding to the outer cell membrane and (2) direct colocalization with GSDMD. This further supports our data showing that TLR2 is not involved, and interaction with the caspase / GSDMD signalling pathway to inhibit IL-33 release (Fig 6D-E). Text has been added to the Results section (lines 163-168) and the Discussion section (209-214).

Significance
This work marks a significant advance in understanding host-microbiome interactions in atopic dermatitis. Identifying commensal-derived lipopeptides as anti-inflammatory mediators challenges the common view of bacterial products as mainly pro-inflammatory. The findings have important implications for developing microbiome-based treatments for AD and potentially other inflammatory skin conditions. Showing that both commensals and S. aureus can produce these protective factors adds complexity to our understanding of the skin microbiome's role in health and disease.
Response: We thank the reviewer for their encouraging appraisal of our manuscript.

Data and Methodology

Strengths:
- Methods are clearly outlined with enough detail for others to reproduce the results.
- Mass spectrometry analysis correctly determines the molecular weight range of the active factors.
- Both chemical inhibitors and genetic approaches (GSDMD-/- mice) are used to validate key findings.
- The data availability statement demonstrates transparency.

Response: Thank you.

Areas for improvement:
- More detailed characterization of native lipopeptide structures using advanced MS/MS or NMR would improve molecular identification.

Response: Please see our detailed responses above, which addresses your comment and that of the Editor. NMR was unfortunately not possible as FSE was a mixture rather than a single structure.

- The ex vivo skin model methods could benefit from additional standardization details.
Response: We have now included extra details in the methods section (lines 328-331) and added a figure panel to provide evidence for the removal of the stratum corneum by tape-stripping (Supplementary Figure 1A).

- Bacterial culture conditions that produce such low OD values should be clarified or re-examined.
Response: Please refer to our response to your comment on "weaknesses" detailed above.

Clarity and Context:
The manuscript is well-written and logically organized. The introduction offers suitable background on AD development and the role of the skin microbiome. The results are presented clearly, starting from initial observations to mechanistic studies. The discussion effectively puts the findings into context within the broader literature and notes limitations. The standardized abbreviations table (Supplementary Table I) improves clarity.
Response: Thank you for your encouraging comments.

References:
The cited literature is thorough and up-to-date. The authors properly reference key studies on AD development, skin microbiome, and GSDMD biology. The discussion of how their findings connect to previous research on S. aureus in AD is well-balanced and recognizes different viewpoints in the field.
Response: Thank you for your encouraging comments.

Suggested Improvements:
1. Structural characterization: Pursue more definitive structural identification of the native bacterial lipopeptides using advanced analytical techniques
Response: Please refer to detailed comment above to the Editor, and to you under "weaknesses".

2. Mechanistic detail: Investigate the receptor(s) or molecular targets through which lipopeptides inhibit GSDMD activation (e.g., TLR2 involvement)
Response: Please see comment detailed above under "*The mechanism connecting lipopeptide binding to GSDMD inhibition needs further clarification*".

3. Dose-response relationships: While dose-response data has been added, expand this to include EC50 values for all active compounds
Response: We have now performed additional dose response experiments (Supplementary Fig. S2). These experiments illustrate that most staphylococcal species have similarly potent immunomodulatory activity down to the limits of the assay. The only exception is *S. carnosus* a staphylococcal species not found on the skin and whose activity is significantly less (Supplementary Fig. S2). The reason for this difference may relate to its higher proliferative activity (as demonstrated in the comparative growth curves in Fig. 1E, insert), and/or its known short-chain C2 acyl groups, now discussed in lines 178-182.

4. Clinical relevance: Discuss potential translation to human studies, including whether these lipopeptides are detectable on human AD skin
Response: A comment regarding translation to human studies is already present in the last paragraph of the Discussion section, including a reference to current studies (Ref. 44) which aimed to determine the potential use of farm dust, which includes bacterial products, in the treatment of allergic airway disease (lines 225-235). We thank the reviewer for their thought provoking-question as to whether bacterial lipopeptides have been detected on human AD skin.

Lipopeptides are integral components of Gram-positive bacterial cell walls and therefore are obviously present on all human healthy and AD skin. However, the authors are unable to find previously published studies demonstrating the presence of free bacterial lipopeptides on human skin. We suggest that it may not be feasible to accurately quantitate free lipopeptides concentrations on the skin surface, as the assay would need to (1) separate free- from membrane-bound bacterial lipopeptides on a surface abounding with bacterial commensals, (2) ensure complete removal of bacteria to exclude the confounding effect of cell wall-bound lipopeptides, and (3) process skin samples without killing the bacteria and triggering shedding of free lipopeptides.

5. Figure improvements:
- Clarify the color coding in all figures (the authors have addressed this for Figure 3)
- Include representative flow cytometry plots or gating strategies in supplementary data
Response: We apologise any confusion. We have now clarified the colour coding in all figures of the manuscript as requested and included a colour key for each graph. We do not have any flow cytometry data in this paper to be able to include flow cytometry plots or gating strategies.

6. Technical clarifications:
- Explain the low bacterial OD values observed
Response: Please refer to our response to the Editor and to you above.

- Provide more details on tape-stripping methodology consistency
Response: As requested, we previously added more information to this part of the Methods section, namely that (1) 8-10 consecutive tape strips using 3.8cm^2 D-Squame® after leaving the tape in place for 10 seconds and that 10^7CFU/ml of S. epi (live *S. epidermidis*) were used. Additional H&E staining on OCT embedded frozen skin sections from this experiment confirming that the stratum corneum is removed following tape stripping have been added to Supplementary Figure 1A.

Overall Assessment
This high-quality manuscript significantly advances our understanding of how commensal bacteria regulate skin inflammation. The work is technically solid, the conclusions are well-supported, and the findings have important implications for AD pathogenesis and treatment. The authors have responded to reviewer feedback and strengthened the manuscript accordingly.
With only minor revisions to address remaining technical issues and improve the structural analysis of the lipopeptides, this work will be ready for publication in Nature Communications.
Response: We thank the reviewer for their encouraging appraisal of our manuscript.

Reviewer #2 (Remarks to the Author):
Most of the previous issues have been addressed. However, I still see concerns with claiming that the treatment works through IL-33. The authors presented data that their *model* is IL-33 dependent, and that FSE reduces IL-33 release in cells. But that does not mean that the treatment is IL-33 dependent. I suppose they could treat the mice with FSE and replace recombinant IL-33 to show that the effects are reduced, but even that is not the same. Without data that the IL-33 levels are reduced in the skin of the mice I don't see how the claims are supported - unless they are arguing that IL-33 is still produced but not signaling. A minimum, the authors should add a paragraph to the discussion outlining that their data is: FSE works through IL33 in cells, the mouse model is IL-33 dependent, but the data for direct action on IL33 in the skin during treatment is lacking.
Response: In response to your comment and as detailed above to the Editor: *We have conducted additional experiments using skin biopsy material from the NC/Tnd mice to address the concern of reviewer 2 regarding IL-33 expression. Results of these new experiments have been added as Fig. 4C &*

4D. Immunofluorescent staining shows that IL-33 expression in the epidermis is markedly suppressed in FSA-stimulated NC/Tnd mice following treatment with both 18h FSE and the synthetic lipopeptide Pam_2CSK_4 compared to mice stimulated with FSA alone. In keeping with our in vitro data using NHEK, we also show that suppression of IL-33 expression by lipopeptides is negated by lipase (LPL) treated 18h FSE (Fig. 4C). Together, these additional results provide further evidence for the role of IL-33 in our animal model of eczema. A comment to this effect has now also been added to the discussion (lines 174-176).

Reviewer #3:
I thank the authors for adequately addressing the open points and for submitting this improved revision.
Response: Thank you.
